# Translational immunoPET imaging using a radiolabeled GD2-specific antibody in neuroblastoma

**DOI:** 10.7150/thno.56736

**Published:** 2022-07-18

**Authors:** Julia Schmitt, Johannes Schwenck, Andreas Maurer, Mirko Przybille, Dominik Sonanini, Gerald Reischl, Jöri E. Wehrmüller, Leticia Quintanilla-Martinez, Stephen D. Gillies, Marcel A. Krueger, Juergen F. Schaefer, Christian la Fougère, Rupert Handgretinger, Bernd J. Pichler

**Affiliations:** 1 Werner Siemens Imaging Center, Department of Preclinical Imaging and Radiopharmacy, Eberhard Karls University Tuebingen, Germany; 2 Department of Nuclear Medicine and Clinical Molecular Imaging, Eberhard Karls University Tuebingen, Germany; 3 Department of Internal Medicine VIII, Eberhard Karls University Tuebingen, Germany; 4 Center for Radiopharmaceutical Sciences ETH-PSI-USZ, Paul Scherrer Institute, Villigen, Switzerland; 5 Institute of Pathology and Neuropathology, University Hospital Tuebingen, Eberhard Karls University Tuebingen, Germany; 6 Provenance Biopharmaceuticals Corp., Carlisle, MA, USA; 7 Department of Diagnostic and Interventional Radiology, Eberhard Karls University Tuebingen, Germany; 8 Childrens Hospital, Department of Hematology and Oncology, Eberhard Karls University Tuebingen, Germany; 9 German Cancer Consortium (DKTK) and German Cancer Research Center (DKFZ), 69120, Heidelberg, Germany; 10 Cluster of Excellence iFIT (EXC 2180) "Image Guided and Functionally Instructed Tumor Therapies", University of Tuebingen, Germany

**Keywords:** neuroblastoma, GD2, PET, ImmuneImaging, theranostic

## Abstract

**Background:** Antibodies targeting surface expressed disialoganglioside GD2 are increasingly used in neuroblastoma immunotherapy and might also have potential for use in radioimmunotherapy. As such targeted treatments might benefit from a dedicated theranostic approach, we studied the influence of radiolabeling on the binding characteristics of ch14.18 antibodies produced by Chinese hamster ovary (CHO) cells and evaluated the benefit of GD2-ImmunoPET as a potential tool for therapy planning.

**Methods:** ^64^Cu was used to reduce radiation burden, which is of high importance especially in a pediatric patient population. ^64^Cu-labeling was accomplished using the chelators NOTA- or DOTAGA-NCS. Radiolabeled antibodies were characterized *in vitro*. [^64^Cu]Cu-DOTAGA-ch14.18/CHO was studied in a neuroblastoma mouse model (subcutaneous CHP-134 xenografts). *In vivo* PET and MR images were acquired at 3 h, 24 h, and 48 h p.i. The specificity of binding was verified using GD2-negative tumors (HEK-293 xenografts), a control antibody and *in vivo* blocking. A first translational application was performed by PET/MRI in a patient with metastasized neuroblastoma.

**Results:** Radiolabeling at an antibody-to-chelator ratio ≥1:10 yielded a product with a radiochemical purity of ≥90% and a specific activity of 0.2-1.0 MBq/µg. Radiochelation was stable over 48 h in PBS, mouse serum or EDTA, and 50.8 ± 3.5% and 50.8 ± 2.0% of the radiolabeled conjugates, prepared at antibody-to-chelator ratios of 1:10 or 1:15, were immunoreactive. *In vivo,* highly specific accumulation (31.6 ± 5.8% ID/g) in neuroblastoma was shown preclinically. Clinical PET/MR scans using [^64^Cu]Cu-NOTA-ch14.18/CHO (NOTA used for safety reasons) could visualize neuroblastoma metastases.

**Conclusions:** *In vivo,* ^64^Cu-labeled ch14.18/CHO is suitable for specific identification of neuroblastoma in PET. A first patient PET indicated the feasibility of the method for clinical translation and the potential utility in image-guided therapy.

## Background

Neuroblastoma (NB) is one of the most frequently occurring extracranial malignancies in early childhood. Although its outcome has improved, cure rates among children with high-risk NB remain poor. Currently, targeted immunotherapy represents a promising approach to improve the prognosis of these patients 1, 2. The disialoganglioside GD2, a tumor-associated antigen that is present in high levels on NB cells and other tumors of neuroendocrine origin, is one of the main targets of antibody-mediated treatments. Several anti-GD2 antibodies have been evaluated for use in immunotherapy 3 and in 2015, the chimeric antibody ch14.18 was approved for the clinical treatment of high-risk NB (Dinutuximab, Unituxin) 4. This antibody, which is produced by SP2/0 murine myeloma cells, is not available in Europe; therefore, the production of ch14.18 in Chinese hamster ovary (ch14.18/CHO) cells was commissioned by the Society of Pediatric Oncology European Neuroblastoma Group (SIOPEN) 5. Production of antibodies by CHO cells has the potential to reduce murine xenotropic retrovirus contaminations and results in an improved glycosylation pattern that helps avoid rapid clearance 5-9. Clinical studies of this antibody were conducted and ch14.18/CHO showed no alterations in clinical activity, toxicity, or pharmacokinetic profile compared to other formats 9, 10. Ch14.18/CHO was approved by the EMA in 2017 (Dinutuximab beta, Qarziba) 11 and even further GD2 directed treatments involving chimeric antigen receptor (CAR) T cells, are currently in clinical development 12.

In addition to immunotherapy, several studies investigated the feasibility of radioimmunotherapy (RIT) with the I-131 labeled GD2-specific antibody 3F8 in NB 13, as well as in medulloblastoma 14 and meningeal metastases 15. As this radioimmunotherapy did not show a survival benefit compared to the treatment with unlabeled 3F8 in metastatic stage 4 NB patients, GD2 targeted RIT is still relatively rarely applied. Instead, radiolabeled metaiodobenzylguanidine (MIBG), which accumulates in catecholaminergic cells, is used in targeted NB radiotherapy ([^131^I]MIBG) 16. As the identical molecule ([^123^I]MIBG) is applied for diagnostic imaging of NB using single photon emission tomography (SPECT), a theranostic approach is available.

A similar theranostic approach, enabling image-guided GD2 targeted therapies, could be beneficial and outperform earlier I-131 GD2 RIT studies 17. Although GD2 is mainly considered to be ubiquitously expressed in NB, a loss either of the complete or partial GD2 expression is described in up to 12% of the cases 18. Furthermore, the tumor uptake of antibodies is also dependent on various other factors like the perfusion or interstitial pressure of the tissue 19. Therefore, theranostic imaging may improve the efficacy of GD2 targeted treatments by potentially allowing an estimation of antibody accumulation in the tumor lesions before start of treatment. Additionally, GD2 imaging could broaden the field of application for GD2 targeted therapy towards tumors with more variable GD2 expression e.g. melanoma or sarcoma and support more exploratory therapeutics like GD2 tumor vaccinations or GD2 CART-cells.

Several GD2 antibodies have already been radiolabeled in recent years. To translate initial SPECT studies 20, 21 to positron emission tomography (PET), investigators have conjugated long-lived positron emitters such as ^64^Cu (half-life 12.7h) to the GD2-specific antibodies hu14.18K322A and ch14.18/SP2/0. These conjugates were tested exclusively in preclinical mouse models 22-24 and therefore additional results are needed for the preparation of a prospective clinical study on GD2-ImmunoPET.

As the therapeutic application of ch14.18 has been approved in the US and in the European Union, there might be high potential to use especially this antibody within a theranostic approach. For this reason, we studied the characteristics of ^64^Cu-labeled ch14.18/CHO 25 *in vitro* and *in vivo* to establish a tool towards PET based planning of GD2 immunotherapy but also to pave the way for the development of a combination of GD2-ImmunoPET with GD2 directed RIT using e.g. Lu-177 within the same construct.

Radiolabeling was based on the bifunctional chelators p-NCS-Bn-NOTA (human study) and p-NCS-Bn-DOTAGA (animal studies). While DOTA is more versatile and also allows the use of other isotopes like Lu-177, NOTA derivatives reportedly yield higher *in vivo* stability for Cu-64 26, 27. Chelator conjugation and radiolabeling of ch14.18/CHO were evaluated in detail to optimize the conjugation protocol for clinical use. Target and off-target accumulation of the radioimmunoconjugate was quantified *in vivo* using immunodeficient CD-1 mice bearing subcutaneous NB xenografts from CHP-134 cells or GD2 negative control tumors from HEK-293 cells. In addition, a first translational approach was performed by assessing GD2 localization using this new radioimmunoconjugate in combined PET and magnetic resonance imaging (MRI), PET/MRI, in a patient suffering from NB before and after GD2 targeted therapy.

## Methods

### Cell Lines

The human GD2-expressing NB cell lines LS and CHP-134 were obtained from DSMZ (Braunschweig, Germany, ACC-675; ACC-653) and GD2-negative human embryonic kidney cells (HEK-293) were purchased from ATCC (Manassas, VA, USA; CRL-1573, CRL-1435) shortly before the study. Both companies characterize cell lines based on short tandem repeat profiles. Throughout the project, the GD2 expression of the cells was verified qualitatively using flow cytometry. All cells were cultured at 37 °C and 5% CO_2_ in RPMI-1640 medium supplemented with penicillin (100 U/mL), streptomycin (100 mg/L), and 10% fetal calf serum (FCS; Biochrom GmbH, Berlin, Germany).

### Antibodies

Ch14.18/CHO was produced by Rentschler Biotechnologie GmbH (Laupheim, Germany) and provided by APEIRON Biologics AG (Vienna, Austria). Antibody used for preclinical studies was for research use only, ch14.18/CHO used for human application was provided in GMP quality for active pharmaceutical ingredients (APIs). The chimeric CD19-specific control antibody 4G7SDIE 27 was produced by Celonics (Basel, Switzerland). Protein concentration was measured photometrically at 280 nm using a Nanodrop ND-1000 photometer (PeQLab, Erlangen, Germany) using IgG settings.

### Chelator Conjugation for Preclinical Studies

Ch14.18/CHO was chelator-conjugated using the chelators p-NCS-Bn-DOTAGA (2-(4,7,10-tris(carboxymethyl)-1,4,7,10-tetraazacyclododecan-1-yl)pentanedioic acid; Chematech, Dijon, France) and p-NCS-Bn-NOTA (2-S-(4-isothiocyanatobenzyl)-1,4,7-triazacyclononane-1,4,7-triacetic acid; Macrocyclics, Plano, TX, USA). During chelator conjugation, antibody-to-chelator ratios (ACR) of 1:15, 1:10, and 1:5 were used for p-NCS-Bn-DOTAGA labeling, and an antibody-to-chelator ratio of 1:15 was used for p-NCS-Bn-NOTA conjugation. For the control antibody 4G7SDIE the same chelators and procedures were used at an ACR of 1:15.

All buffers were treated with 1.2 g/L Chelex 100 (sodium form, Sigma-Aldrich, St. Louis, MO, USA) to avoid contamination with metals. For removal of metal contaminations ch14.18/CHO was pretreated with 2.8 mM EDTA for 30 min at room temperature (RT).

For p-NCS-Bn-DOTAGA conjugation, the buffer was exchanged to 0.1 M sodium bicarbonate pH 9 using Amicon Ultra-15 centrifugal filter units (three rounds of filtration) with a molecular weight cutoff of 30 kDa (Merck KGaA, Darmstadt, Germany). The chelator was dissolved in water at 10 mg/ml and added to the antibody (7.5 mg/ml, 440 µl per reaction) at the respective ACR. The conjugation reaction was conducted at RT for 1 h. Subsequently, the antibody was purified from excess chelator and buffered with 0.25 M sodium acetate pH 6 using an Amicon Ultra-15 centrifugal filter unit (seven rounds of filtration). Antibody integrity was measured before and after conjugation by HPSEC. The details of p-NCS-Bn-NOTA conjugation clinical tracer production are described in the respective section.

The number of chelators per antibody was assessed by mass spectrometry 28. Briefly, the antibody samples were deglycosylated by overnight incubation with 1 U PNGase F (Hoffmann-La Roche AG, Basel, Switzerland) at 37 °C and subsequently reduced by the addition of 50 mM DTT (Sigma-Aldrich) and incubation for at least 10 min at 37 °C prior to chromatographic separation and mass spectrometry using electrospray ionization (ESI) on a LCT Premier mass spectrometer (Waters, Milford, MA, USA). Liquid chromatography (LC) was performed at a column temperature of 80 °C on an Aeris WIDEPORE XB-C18 column (3.6 μm, 100 mm x 2.1 mm; Phenomenex, Torrance, CA, USA) and a gradient of water, acetonitrile, and isopropanol over 20 min.

The obtained spectra were analyzed using MassLynx V4.1 and deconvoluted using the MaxEnt1 algorithm. The conjugation ratio *R_c_* was calculated as *R_c_* = *I_c_* / (*I_c_* + *I_nc_*) x 100% with *I_c_* corresponding to the intensity of the conjugated and *I_nc_* of the unconjugated protein.

### Radiolabeling

^64^Cu was produced in a PETtrace cyclotron (16 MeV; GE Medical Systems, Uppsala, Sweden) by proton irradiation of ^64^Ni on a platinum/iridium plate (90/10) (30 mg, > 95% enrichment; Chemotrade Chemiehandelsgesellschaft mbH, Duesseldorf, Germany) and separated from metallic impurities by ion-exchange chromatography (AG1x8, Bio-Rad Laboratories, Hercules, CA, USA). Radiolabeling was performed according to a modification of a procedure that has been described in the literature 29. Briefly, chelator-conjugated antibodies were incubated with 0.2-1 MBq [^64^Cu]CuCl_2_ per µg for 1 h at 42 °C in 0.25 M sodium acetate buffer. Quality control was performed by thin-layer chromatography (TLC) using Polygram SIL G/UV_254_ plates (Macherey-Nagel, Dueren, Germany) and citrate buffer (tracer remained at baseline, observed Rf for unbound ^64^Cu of 0.2-0.8). High-performance size-exclusion chromatography (HPSEC, BioSep SEC-s3000, Phenomenex; saline sodium citrate buffer, 1 ml/min) was used to check for fragmentation and aggregation of the antibodies.

To assess the release of ^64^Cu from the radiotracer, the radioimmunoconjugates were incubated with PBS, mouse serum (Life Technologies, Carlsbad, CA, USA), or in presence of a competitor (ethylenediaminetetraacetic acid (EDTA); Sigma-Aldrich) at 37 °C for a period of 2 days. TLC was performed repetitively (n = 1-3).

### *In vitro* Studies

For *in vitro* experiments, adherent cells were detached using trypsin (Biochrom) and manually counted in a Neubauer-counting chamber after staining with trypan blue (Bio-Rad Laboratories). Neuroblastoma (CHP-134 or LS) or HEK-293 cells (1x10^6^ cells) were incubated in triplicate with 100 ng (ca. 67 kBq) of the radiolabeled antibody for 1 h at 37 °C. After the cells had been washed twice with PBS containing 3% bovine serum albumin (BSA; Sigma-Aldrich), the radioactivity in the cell pellets was determined by gamma counting using a Wizard^2^ 2480 gamma counter (PerkinElmer Inc., Waltham, MA, USA) (n = 3-4, each in triplicate). Blocking studies were conducted by adding 10 µg of unconjugated antibody to the cells 30 min prior to incubation of the cells with the radiolabeled antibody (n = 4, each in triplicate).

The immunoreactivity of [^64^Cu]Cu-DOTAGA-ch14.18/CHO was quantified using an assay developed by Lindmo *et al.* 30. Briefly, increasing numbers of GD2-expressing LS cells (0.5-15 x 10^6^) were incubated with a constant amount of antibody (50-100 ng) as described above. By linear curve fitting of double inverse plots of 1/bound activity as a function of 1/cell count, the immunoreactive fraction (IF) was calculated (y-intercept; n = 3-4 per antibody). Nonspecific binding was measured using GD2-negative cells.

### Xenograft Models

All animal experiments and housing conditions were approved by the regulatory authorities (Regierungspraesidium Tuebingen; R10/16). Six-week-old CD1 nude mice (female) were purchased from Charles River Laboratories (Sulzfeld, Germany). The animals were housed in our vivarium under a 12/12 h light/dark cycle and were provided with food and water *ad libitum*. For tumor inoculation, 6 x 10^6^ HEK-293 or CHP-134 cells in 50% Matrigel (BD Biosciences, Franklin Lakes, NJ, USA) were subcutaneously injected into the animals' flanks. Experiments were performed when the tumors had reached a size of approximately 1 cm³.

### Preclinical* in vivo* Studies

The *in vivo* biodistribution of the radiolabeled antibodies was assessed using an Inveon small-animal PET scanner (Siemens Preclinical Solutions, Knoxville, TN, USA). [^64^Cu]Cu-DOTAGA-labeled antibody (12.25 ± 0.64 MBq, ACR 1:10) was injected intravenously into tumor-bearing mice (HEK-293+ch14.18/CHO: n = 7; CHP-134+ch14.18/CHO: n = 7; CHP-134+4G7SDIE: n = 7). Static 10 min PET scans and corresponding T2-weighted MR measurements were performed successively 3 h, 24 h, and 48 h after ^64^Cu-antibody injection (50 µg). For blocking, 500 µg of unconjugated ch14.18/CHO was injected 24 h prior to [^64^Cu]Cu-DOTAGA-antibody administration (HEK-293+ch14.18/CHO: n = 4; CHP-134+ch14.18/CHO: n = 8; CHP-134+4G7SDIE: n = 4).

During measurements, the mice were anesthetized with 1.5% isoflurane (CP-Pharma Handelsgesellschaft mbH, Burgdorf, Germany) evaporated in isoflurane + oxygen mixture. The depth of anesthesia was monitored by breath frequency. MR measurements were performed immediately after the PET scans by transferring the animals on their beds to a 7-Tesla small animal MRI scanner (Clinscan, Bruker BioSpin GmbH, Rheinstetten, Germany). Anatomical structures were visualized by T2-weighted imaging consisting of a 3D-spoiled turbo spin echo sequence (256 x 161 matrix, 35 x 57 mm^2^ FOV, TR = 3000 ms, TE = 205 ms, ST = 0.22 mm).

PET images were reconstructed using an ordered subset expectation maximization (OSEM-2D) algorithm. After manual fusion of reconstructed PET and MR images, volumes of interest were contoured based on MR information and tracer accumulation was analyzed using Inveon Research Workplace software (Siemens Preclinical Solutions). The results are expressed as percentage of the injected dose per volume (% ID/cc), tumor-to-muscle ratio (TMR), and tumor-to-liver ratio (TLR). After final imaging, an *ex vivo* biodistribution analysis was performed using the gamma counter. Decay-corrected radioactivity was normalized to organ weight: the results are expressed as % ID/g, TMR, TLR, and tumor-to-blood ratio (TBR).

### Tissue Staining

After the last PET scan, tumor and muscle tissues were frozen in Tissue-Tek O.C.T. Compound (Sakura, Zoeterwonde, Netherlands), and 20 µm-thick cryosections were exposed to a phosphor screen (445SI, Molecular Dynamics, Sunnyvale, CA, USA) for 24 h. The autoradiographic results were read using a Storm 840 phosphorimager (Amersham Biosciences, Amersham, UK) and evaluated using ImageJ (National Institutes of Health, Bethesda, MD, USA). For comparison of different studies TMR was quantified. Following the autoradiography, the cryosections were stained with hematoxylin and eosin (H&E).

Additional tumors were fixed in formalin and embedded in paraffin (SAV-Liquid Production GmbH, Flintsbach, Germany). For morphological analysis, 3-5 µm sections were cut and stained with H&E. Immunohistochemistry was performed on an automated immunostainer (Discovery XT, Ventana Medical Systems, Inc., Tucson, AZ, USA) according to the manufacturer's protocols for open procedures. Sections were stained with CD31 antibody (Abcam, Cambridge, UK). To detect the primary chimeric antibody, tumor sections from the PET-scanned animals were stained using a secondary biotinylated anti-human IgG antibody (Thermo Scientific, Waltham, MA, USA) 48 h after antibody injection. GD2 expression in the tumor tissue was verified using the GD2-specific antibody 14G2a (Merck KGaA, dilution 1:100, 32 minutes incubation). Appropriate positive (glioblastoma) and negative controls (GD2 negative tumor (HEK-293)) were used to confirm the adequacy of the stainings. All slides were scanned with a NanoZoomer 2.0 HT digital slide scanner and the scans were evaluated using the software NDP.view (Hamamatsu Photonics K.K., Hamamatsu, Japan).

### Radiopharmaceutical Preparation for Human Application

Preparation of the radiopharmaceutical for human application was performed under direct responsibility of the nuclear medicine physician within the framework of article §13 2b of the german medicinal products act (“Arzneimittelgesetz”; AMG) in a good manufacturing practice (GMP) facility. The antibody was conjugated using p-SCN-Bn-NOTA as chelator. Conjugation was performed in a manner similar to that used in the DOTAGA conjugation described above for the preclinical experiments and the procedure reported in the literature 31. Briefly, 20 mg of ch14.18/CHO was buffer-exchanged to 0.1 M 4-(2-hydroxyethyl)-1-piperazineethanesulfonic acid (HEPES) pH 8.9 after incubation of the antibody with 2.5 µmol of EDTA for 30 min at room temperature. p-SCN-Bn-NOTA was dissolved in DMSO and added to the antibody at 15x molar excess followed by overnight incubation at 4 °C. After washing with 0.1 M sodium acetate pH 6, the protein solution was characterized with respect to protein concentration, HPSEC profile, chelator number, bioburden, endotoxins, radiolabeling properties (validation syntheses), and other parameters required for stability analysis and patient safety.

For radiolabeling, the antibody was incubated with a buffered solution of ^64^Cu (0.5 MBq per µg) in a procedure similar to that used for radiolabeling for preclinical use as described above. After radiolabeling, 1 µL of 20% Ca-diethylenetriaminepentaacetic acid (DTPA; Heyl, Berlin, Germany, injection solution for human use) was added to the tracer solution followed by dilution with PBS and sterile filtration (final composition: active pharmaceutical ingredients: [^64^Cu]Cu-NOTA-ch14.18/CHO; NOTA-ch14.18/CHO; excipients: water for injection, ammoniumacetate, calcium-trisodium-pentetate (DTPA, diethylenetriaminepentaacetic acid), sodium chloride, sodium dihydrogenphosphate, disodium hydrogenphosphate). Quality control samples were analyzed for appearance, identity, chemical and radiochemical purity (RCP) (TLC, HPSEC), pH, radionuclidic purity, endotoxins, and sterility (retrospectively): all tests were performed according to pharmacopoeia methods. Radiolabeled samples were tested *in vitro* for target binding and stability using the methods described above.

### Clinical Imaging

Following the stipulations of the AMG §13(2b) a first-in-human PET/MRI scan with [^64^Cu]Cu-NOTA-ch14.18/CHO was performed in a 6 y/o NB patient suffering from bone metastases and diffuse bone marrow infiltration. The patient was referred to the experimental diagnostics unit by his pediatric oncologist, who was facing an unmet diagnostic challenge that could not be solved sufficiently using standard diagnostic means. The parents of the child participant provided written informed assent to the procedure on the child's behalf.

After bone marrow transplantation, the patient received three cycles of immunotherapy with 16-17 mg of ch14.18/CHO at intervals of 4-6 weeks.

One day prior to the first immunotherapy cycle and 18 days after the completion of the third cycle, both combined PET/MRI examinations were performed 24 h p.i. of 78 MBq [^64^Cu]Cu-NOTA-ch14.18/CHO (156 µg antibody, 3.7 MBq per kg body weight) on a whole-body PET/MRI system (Biograph mMR, Siemens Healthineers, Erlangen, Germany). PET images (6 min acquisition per bed position, 6 bed positions) were reconstructed using an OSEM-3D algorithm and corrected for scatter and attenuation using standard MR-based segmentation as described previously 32-35. A coronal T1-weighted 3D-encoded spoiled gradient-echo sequence with double-echo for Dixon-based fat-water separation for attenuation correction, a coronal T2 turbo inversion recovery magnitude (TIRM), and transverse diffusion-weighted (DWI) echo-planar imaging sequences were acquired simultaneously to PET imaging.

[^123^I]MIBG SPECT/CT examinations were acquired 24 h p.i. of the radiotracer (96.9 and 101.4 MBq) according to clinical standards on a Discovery 670 Pro (GE Healthcare, Chicago, USA) using a medium energy general purpose collimator. The SPECT images were reconstructed using an OSEM algorithm with 2 iterations and 10 subsets. Additionally, attenuation and scatter correction as well as resolution recovery were applied. SPECT/CT examinations were acquired 5 weeks before the first cycle of ch14.18/CHO therapy (baseline) and 2 days (follow-up) after the completion of the third cycle.

### Statistical Analysis

Statistical analysis was conducted using a two-sided Student's t-test; multiple-group comparisons were performed using a Tukey-Kramer test in Origin 8 software (OriginLab Corporation, Northampton, MA, USA). The data were considered significantly different when p ≤ 0.05. All quantitative results are presented as the mean ± 1 standard deviation.

## Results

### Radiolabeling

A schematic of the radiolabeling procedure is shown in Figure S1. Chelator-conjugated antibodies had an unchanged HPSEC elution profile as compared to the unconjugated antibody (Figure S2). Chelator-to-antibody ratios of 0.9, 1.2, and 1.4 were obtained for ch14.18/CHO conjugates to DOTAGA at ACRs of 1:5, 1:10, and 1:15 (Figure S3). For the DOTAGA-conjugated antibody, an RCP of ≥90% was routinely achieved when an ACR ≥1:10 was used. At smaller ACRs, unreliable labeling performance was observed depending on the quality of the ^64^Cu batch (DOTAGA 1:5 only up to 70%).

Stable radioconjugation was observed following incubation of the radioimmunoconjugates (DOTAGA-conjugated in at ACRs of 1:15 or 1:10) in PBS, serum, or EDTA. After 48 h, the radioconjugates retained > 80% of the original RCP in all buffers.

### *In vitro* Studies

**Figure 1:**
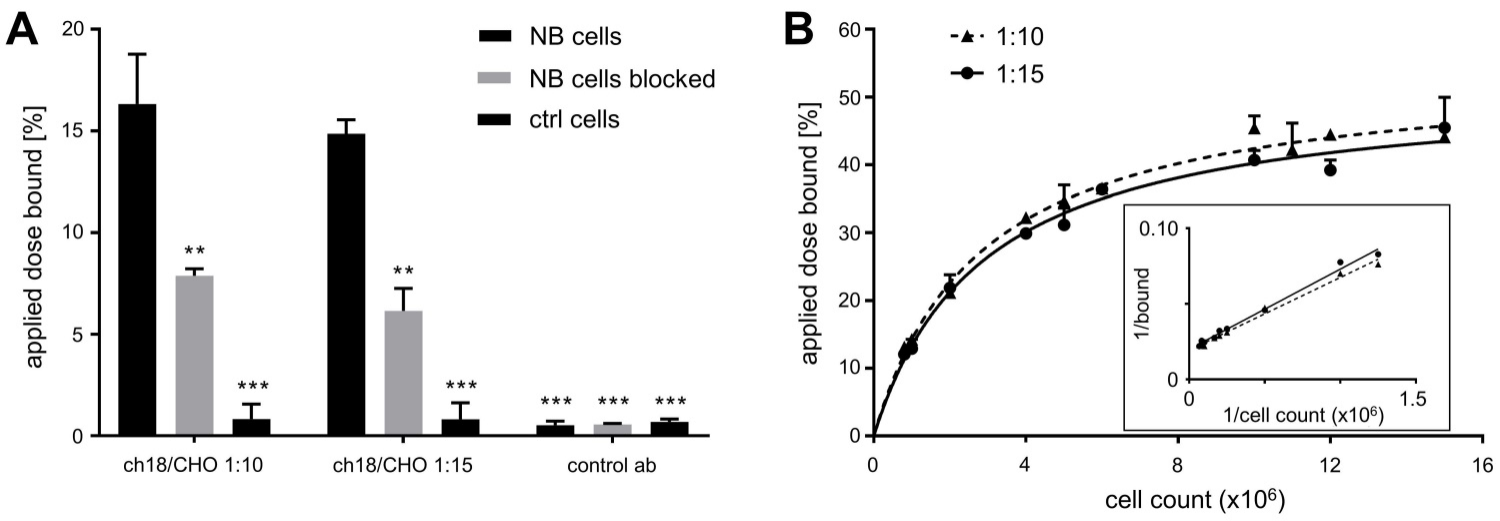
***In vitro* characteristics of ^64^Cu-labeled ch14.18/CHO.** As determined by gamma counter studies, GD2-expressing cells (LS and CHP-134) showed strongly increased binding of [^64^Cu]Cu-DOTAGA-ch14.18/CHO (conjugated in ACRs of 1:10 or 1:15) compared to controls (GD2-negative cells or a nonspecific antibody). Blocking of GD2 led to a great reduction in the cell-associated binding of the ch14.18/CHO-based radioimmunoconjugates (ACRs 1:10, 1:15), thereby demonstrating their specificity (A). The amount of radioactivity associated with cells in reference to the total activity applied is shown. ** and *** indicate significant differences in the NB cell surface association of [^64^Cu]Cu-DOTAGA-ch14.18/CHO at p < 0.01 and p < 0.001, respectively. The conventional plot of a Lindmo binding assay displays the ratio of bound to total applied radioactivity as a function of cell concentration (B). In the double inverse plot, a fitted line through the data points determines the IF of the antibodies conjugated with DOTAGA-NCS at ACRs of 1:10 and 1:15 (insert B, IFs: 1:10: 50.8 ± 3.5%; 1:15: 50.8 ± 2.0%).

In a gamma counter assay, GD2-expressing cells (pooled data of CHP-134 and LS cells) exhibited strongly increased uptake of [^64^Cu]Cu-DOTAGA-ch14.18/CHO prepared at ACRs of 1:10 or 1:15 in comparison to controls (HEK-293 cells, control antibody: p < 0.001, Figure 1A). Blocking GD2 significantly reduced the cell-associated radioactivity for both GD2-specific radioimmunoconjugates (p < 0.01, Figure 1A).

In the Lindmo-assay the measured IFs of the radioimmunoconjugates were 50.8 ± 3.5% and 50.8 ± 2.0% for ch14.18/CHO-DOTA conjugates prepared at labeling ratios of 1:10 and 1:15, respectively (Figure 1B).

Due to the insufficient radiolabeling yield in combination with a lack of relevant increase in IF, ch14.18/CHO conjugated with an ACR of 1:5 was not analyzed further.

### Preclinical* in vivo* PET Imaging

**Figure 2:**
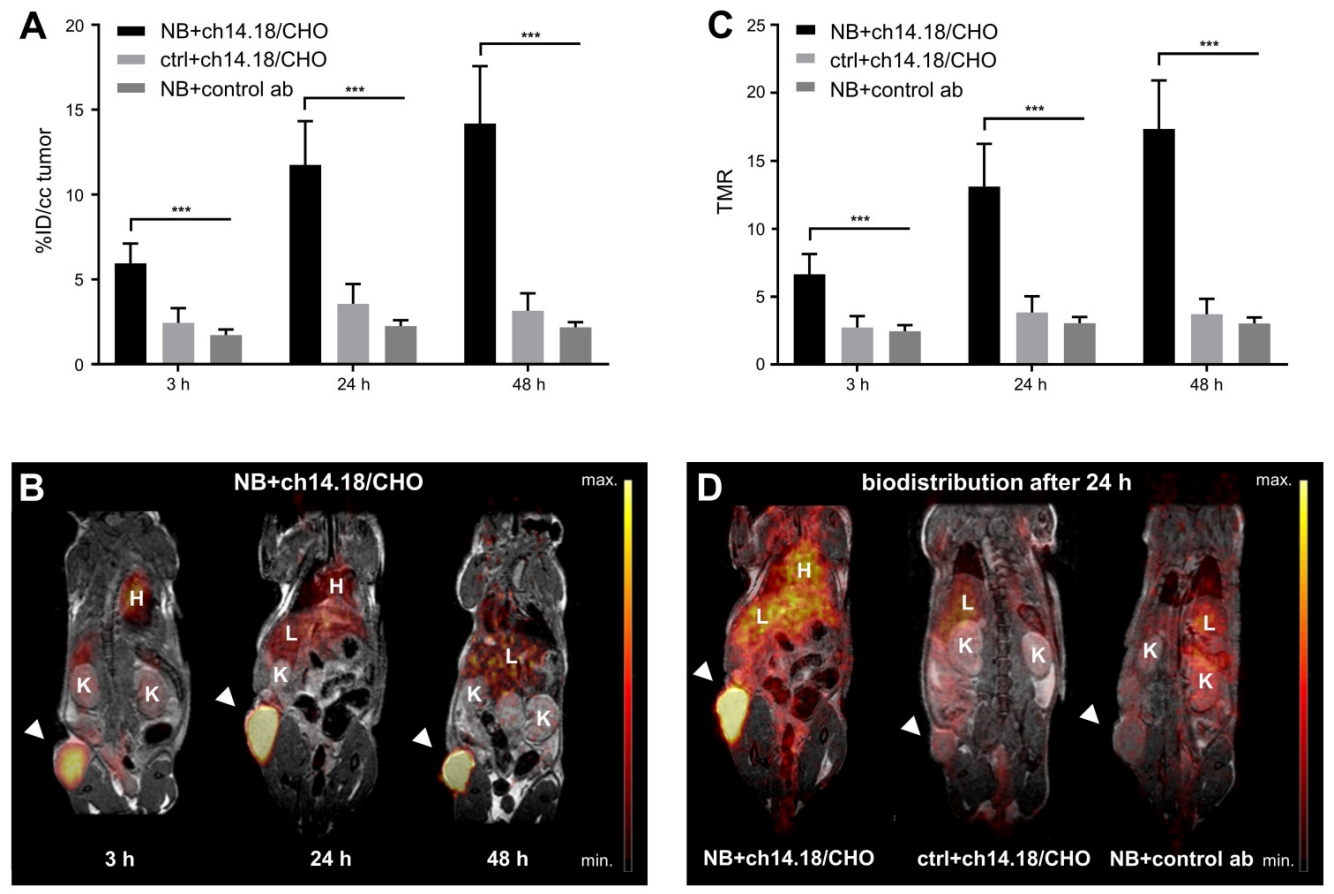
***In vivo* PET imaging: Biodistribution of radiolabeled ch14.18/CHO.** *In vivo* tumor uptake of [^64^Cu]Cu-DOTAGA-labeled ch14.18/CHO and of the radiolabeled control antibody (control ab) is shown in % ID/cc for the three imaging time points and the tumor models CHP-134 (NB) and HEK-293 (ctrl) (A). Whole-body PET images display the distribution of the tracer at 3 h, 24 h, and 48 h after injection of radiolabeled ch14.18/CHO into NB-bearing mice (B). TMR for the same imaging time points is shown in (C). PET images acquired 24 h after tracer injection show the *in vivo* accumulation of [^64^Cu]Cu-DOTAGA-ch14.18/CHO in NB in comparison with its uptake into control lesions and with the accumulation of the radiolabeled control antibody in NB (D). Tumors are indicated by arrows. Liver (L), heart (H) and kidneys (K) are indicated as reference. Significant differences at p < 0.001 are indicated by ***.

**Figure 3:**
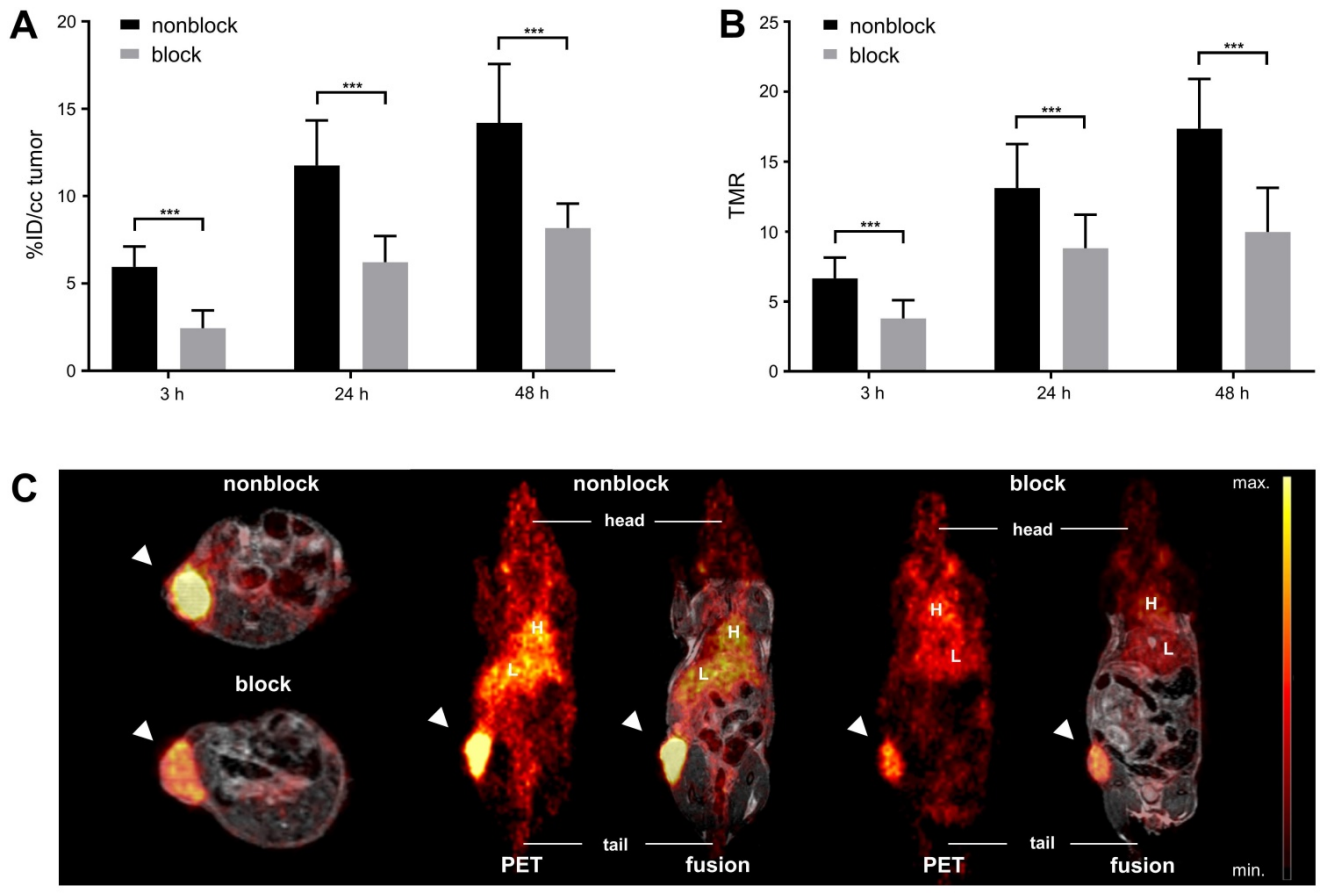
***In vivo* blocking study.** *In vivo* NB uptake of [^64^Cu]Cu-DOTAGA-labeled ch14.18/CHO is shown in % ID/cc for animals that did or did not receive an injection of 500 µg unlabeled ch14.18/CHO 24 h prior to tracer administration to block GD2 (A). TMR is depicted for the same groups (B); both results indicate highly specific binding of [^64^Cu]Cu-DOTAGA-ch14.18/CHO *in vivo*. PET and MR images acquired 24 h after tracer application show reduced accumulation of the tracer in tumors after blocking (C). Tumors are indicated by arrows. Liver (L) and heart (H) are indicated for reference. Significant differences at p < 0.001 are indicated by ***.

Over the time course of the *in vivo* PET study (Figure 2), [^64^Cu]Cu-DOTAGA-ch14.18/CHO reached a maximum tumor uptake of 14.2 ± 3.4% ID/cc and a TMR of 17.4 ± 3.6 48 h after tracer injection (Figure 2A/C). In comparison to GD2-negative tumors, NB uptake of ch14.18/CHO and its TMR was significantly higher at all time points examined (p < 0.001). Furthermore, accumulation of radiolabeled ch14.18/CHO in NBs significantly exceeded uptake of the radiolabeled control antibody, and its TMR was significantly higher (3 h, 24 h, 48 h: p < 0.001). *In vivo* blocking significantly reduced tumor uptake of [^64^Cu]Cu-DOTAGA-ch14.18/CHO to 2.4 ± 1.0% ID/cc, 6.2 ± 1.5% ID/cc, and 8.2 ± 1.4% ID/cc after 3 h, 24 h, and 48 h (p < 0.001, Figure 3). Furthermore, TLR (3 h, 24 h: p < 0.001, data not shown) and TMR (3 h, 24 h, 48 h: p < 0.001, Figure 3B) were decreased significantly.

**Figure 4:**
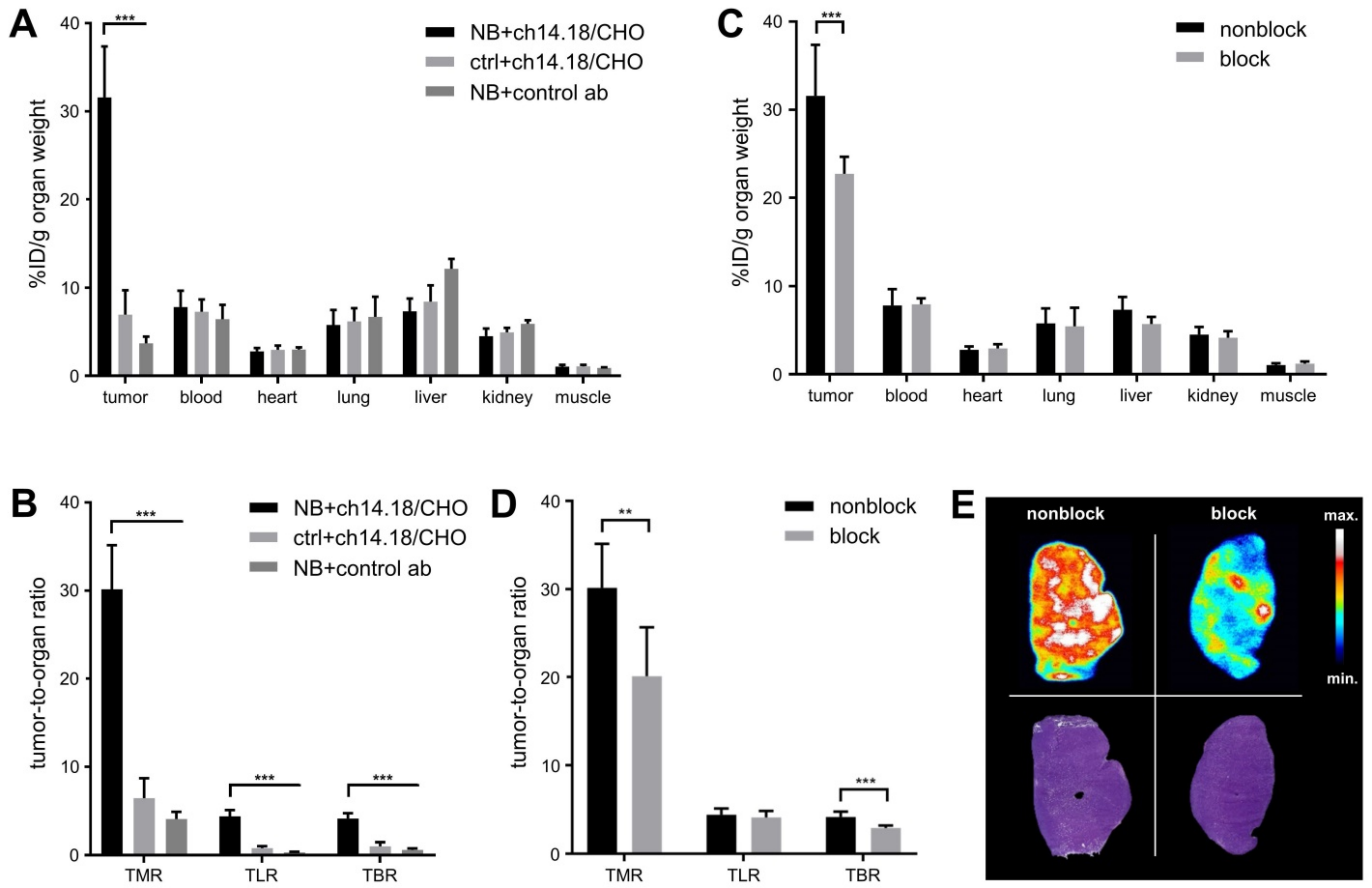
***Ex vivo* biodistribution.** At 48 h after *in vivo* tracer uptake, the biodistribution of [^64^Cu]Cu-DOTAGA-ch14.18/CHO and that of a radiolabeled control antibody (control ab) in diverse organs *ex vivo* were quantified using a gamma counter. Uptake values are given in % ID/g for NB and HEK-293 tumor-bearing animals (A) and for NB-bearing animals after blocking GD2 with unlabeled ch14.18/CHO (C). The results verified the specificity of the GD2 targeting tracer. The tumor-to-organ ratios TMR, TLR, and TBR observed in both studies are shown (B/D). Autoradiography (upper panel) supported the findings, H&E stainings of the autoradiography slides are shown for comparison (lower panel); a representative animal from the blocking study is shown (E). Significant differences at p < 0.01 or p < 0.001 are indicated by ** or ***, respectively.

The *ex vivo* biodistribution corresponded to an accumulation of [^64^Cu]Cu-DOTAGA-ch14.18/CHO of 31.6 ± 5.8% ID/g in CHP-134 NBs with a TMR of 30.2 ± 5.0 (Table 1, Figure 4A/B). The background accumulation in other organs remained below 8% ID/g. TLR was 4.4 ± 0.7, and TBR was quantified as 4.1 ± 0.6. [^64^Cu]Cu-DOTAGA-ch14.18/CHO showed a significantly lower accumulation of 6.9 ± 2.8% ID/g (p < 0.001) in GD2-negative tumors as well as significantly higher uptake in NB compared to the radiolabeled nonspecific control antibody (3.7 ± 0.7% ID/g, p < 0.001). After injection of 500 µg blocking antibody, tumor uptake (22.7 ± 2.0% ID/g) and TMR (20.1 ± 5.6) of the GD2-specific radioimmunoconjugate were significantly reduced (p < 0.005, Table 1, Figure 4C-E).

**Figure 5:**
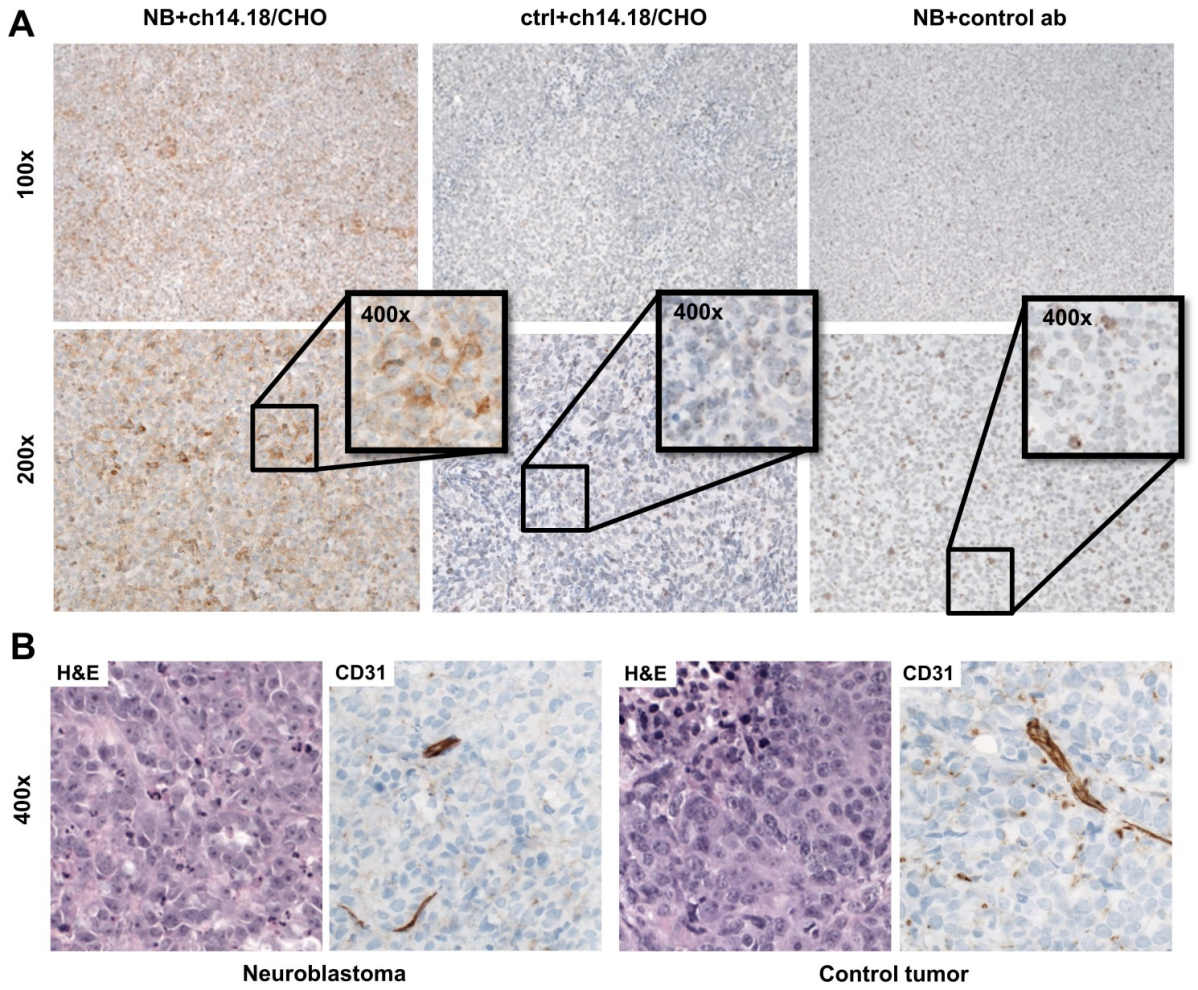
**Histology and immunohistochemistry of xenografts.** Immunohistochemical staining of tumor sections revealed specific uptake of [^64^Cu]Cu-DOTAGA-ch14.18/CHO in NBs 48 h after tracer injection (A). After injection of [^64^Cu]Cu-DOTAGA-ch14.18/CHO into NB-bearing animals, tumors showed clearly positive staining (left panel). Controls (GD2-negative tumors; ctrl and control antibody; ab) showed only a slight background signal (middle and right panels). H&E staining of CHP-134 and HEK-293 lesions revealed large cells with irregular nuclei, open chromatin and prominent nucleoli. CD31 immunostaining indicated blood vessels of variable caliber in both xenografts (B).

Autoradiography of tumor (Figure 4E) and muscle as well as immunohistochemical staining to determine antibody distribution in the tumors (Figure 5A) supported these findings. After injection of 50 µg radiolabeled ch14.18/CHO, the NB xenografts demonstrated high signal intensity in autoradiography, and with immunohistochemistry clearly specific GD2 membrane staining was distributed through the whole tumor. The autoradiographic signal intensity was greatly reduced after blocking (Figure 4E). After [^64^Cu]Cu-DOTAGA-4G7SDIE injection, CHP-134 tumor slides showed only a low autoradiographic signal (Figure S4) and nonspecific background staining in immunohistochemistry (Figure 5A). HEK-293 tumor sections showed only background radioactivity signal (Figure S4) and staining (Figure 5A). GD2 expression was only observed in NBs (Figure S5). H&E and CD31 staining demonstrated the comparability of the tumors (Figure 5B). H&E staining of CHP-134 NBs and HEK-293 lesions showed large cells with irregular nuclei, open chromatin, and prominent nucleoli. CD31 immunostaining revealed blood vessels of variable caliber in both xenografts.

### Preparation for Human Application

Although the preclinical imaging results were promising despite the use of a suboptimal chelator for ^64^Cu (DOTAGA), we decided to change the chelator to NOTA, which has a higher affinity to Cu. Especially in pediatric patients this minimizes the risk of unintended radiation exposure e.g. by free ^64^Cu uptake in the liver, kidneys or the bone marrow. This is also important in regards to the preparation of a prospective study investigating the diagnostic value of GD2-PET in Neuroblastoma patients.

Following the conjugation protocol described above, LC-MS analysis displayed successful conjugation of ca. 2.3 chelator molecules per antibody (Figure S3). Radiolabeling consistently yielded radiochemical purities of > 95% with a set specific radioactivity of 0.5 MBq/µg. All QC results met the specifications corresponding to the pharmacopeia requirements for radiopharmaceuticals. The radiochelation of NOTA-ch14.18/CHO was shown to remain consistent (RCP: 89.7%; 90.4%; 87.8% at 48 h post labeling after 2 days of incubation in PBS, mouse serum, or 2 mM EDTA; Figure S6). Furthermore, [^64^Cu]Cu-NOTA-ch14.18/CHO showed GD2-specific binding *in vitro* (Figure S6).

### Clinical Imaging

**Figure 6:**
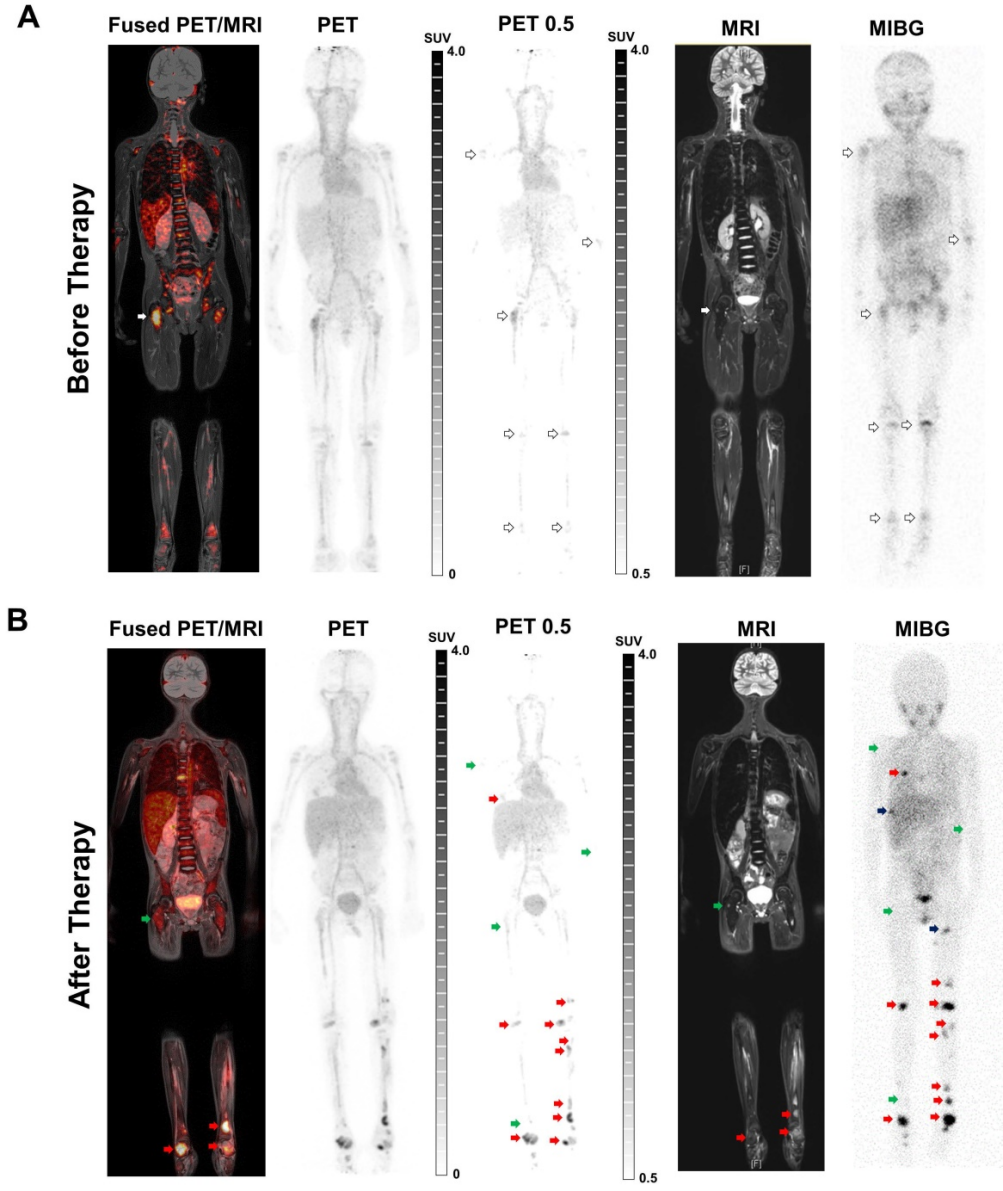
** Clinical GD2-specific ImmunoPET/MRI of Neuroblastoma.** First-in-human GD2-PET/MR scans were performed after application of ^64^Cu-NOTA-ch14.18/CHO in a 6 y/o patient suffering from metastatic neuroblastoma prior to and after therapeutic application of ch14.18/CHO. To better distinguish between metastasis and the unspecific uptake in the bone (especially in the spine and the pelvis) the windowing was adapted to a lower threshold of SUV 0.5 in order to minimize the visible background signal (PET 0.5; see Figure S7A). A: Positive ^64^Cu-NOTA-ch14.18/CHO foci were found in several lesions (as indicated by arrows) on coronal fused PET/MRI and PET-MIP images. Simultaneous MRI (whole-body T2 TIRM) and planar [^123^I]MIBG scintigraphy (five weeks prior to GD2-PET/MRI) revealed diffuse bone marrow infiltration as well as bone metastases corresponding to the GD2-positive lesions. B: Follow-up GD2-PET/MRI 2.5 weeks after the 3^rd^ therapeutic application and 3 months after initial PET/MRI indicated a mixed response to anti-GD2 therapy. Treatment response was observed in some lesions (green arrows), e.g., in the right femur. In addition, several progressive as well as new bone metastases (red arrow, exemplary lesions) were detected after therapy. MRI (whole-body T2 TIRM) and planar [^123^I]MIBG scintigraphy (2.5 weeks before GD2-PET/MRI, 2 days after completion of the third cycle) confirmed progressive disease but also identified additional lesions (examples, black arrow) that were not detected by GD2-PET.

One day before the first cycle of immunotherapy with ch14.18/CHO, [^64^Cu]Cu-NOTA-ch14.18/CHO PET (GD2-PET) indicated tracer accumulation in several lesions in a patient with metastatic NB. PET images most likely showed also an unspecific and perfusion-related tracer deposition in the blood pool, as well as in unaffected organs and bone marrow (especially in the spine; Figure S7). Thus, for better visualization of NB metastases and differentiation of the metastases from the unspecific uptake in bone marrow, an adapted windowing of the PET images was used, whereby the windowing was adjusted to a lower threshold of SUV 0.5 in order to minimize the visible unspecific background signal (PET 0.5; Figure 6A/B).

PET images revealed the highest uptake of the radiolabeled antibody in the right femur (Figure 6A left panel, white arrows). Corresponding MRI and [^123^I]MIBG SPECT/CT (Figure 6A right panel) scans performed 5 weeks prior to PET identified lesions corresponding to the findings in GD2-PET. Furthermore, [^123^I]MIBG SPECT/CT revealed an additional diffuse bone marrow infiltration especially in the pelvis and in the lower extremities which were only hinted provable in GD2-PET after an adapted windowing was applied (PET 0.5).

In the follow-up GD2-PET performed 18 days after the third therapeutic cycle of ch14.18/CHO antibody, some of the lesions showed reduced accumulation of the radiolabeled antibody compared to the baseline scan (Figure 6B left panel, green arrows); in particular the uptake in the femoral lesion was demonstrably reduced. The same lesions indicated treatment response according to MRI and [^123^I]MIBG findings (Figure 6B right panel, green arrows). However, additional new bone metastases were detected in the second GD2-PET (Figure 6B left panel; red arrow; exemplary lesions), which were confirmed by MRI (Figure 6B right panel; whole-body TIRM) and planar [^123^I]MIBG scintigraphy (Figure 6B; right panel). Though, some new bone lesions that were indicated by [^123^I]MIBG scintigraphy and MRI were not detectable by GD2-PET (Figure 6B, black arrows). The diffuse bone marrow infiltration in the [^123^I]MIBG SPECT/CT was almost completely diminished after therapy, while still detectable antibody uptake was found in the bone marrow in GD2-PET suggesting potential the bone marrow infiltration (e.g. femur and tibia, both sides).

## Discussion

The use of GD2-specific antibodies in NB immunotherapy has been studied extensively and a few antibodies have already found their way into clinical routine 3, 22, 23, 36. Similar to most other targeted therapies, a major challenge of current GD2 targeting immunotherapies is the individual response to therapy and the lack of adequate predictive markers 37. In this regard, radiolabeled antibodies have a high potential as they might be applied for guidance of these complex therapeutic approaches using the same antibody for imaging and subsequent (radio)immunotherapy. Recently, Bensch et al. impressively demonstrated the potential of such a theranostic approach by correlating the accumulation of a radiolabeled antibody against programmed death ligand 1 (PD-L1) in tumors with the therapeutic response to immune checkpoint blockade and showed the superiority of this method to standard histological evaluation of PD-L1-expression in the initial tumor biopsy 38.

Here, we studied the *in vitro* and *in vivo* characteristics of ^64^Cu-labeled ch14.18 produced in CHO cells (Dinutixumab beta) to establish a tool towards PET based planning of GD2 directed immunotherapy but also to pave the way for the development of a theranostic combination of ^64^Cu imaging and GD2 targeting radioimmunotherapy within the same construct. GD2 imaging could furthermore broaden the field of application for GD2 directed therapy towards tumors with more variable GD2 expression e.g. melanoma or sarcoma and support more exploratory therapeutics like GD2 tumor vaccinations or GD2 CART-cells.

After a thorough preclinical *in vitro* and *in vivo* evaluation of the benefit of ^64^Cu-labeled ch14.18/CHO for NB diagnostics, a first translational approach involving its compassionate use in an individual patient suffering from NB was performed. We decided to use ^64^Cu, instead of e.g. ^89^Zr as radiolabel for the anti-GD2 antibody in order to keep the radiation dose for the patients low, which is of high importance especially in a pediatric patient population 39. We believe, ^64^Cu still allows sufficient uptake time, specifically if the images are intended to be applied for treatment planning.

Our data demonstrate the feasibility of ^64^Cu-labeling of the GD2-specific antibody ch14.18/CHO using the chelators DOTAGA-NCS and NOTA-NCS. Although chelator conjugation and radiolabeling were conducted under mild conditions and we achieved moderate chelator numbers per antibody, the IF of the radiolabeled GD2-specific antibodies were below 60%. In the applied assay set-up, the apparent IFs might be influenced by rapid shedding of GD2 from target cells, leading to blockage of the radiolabeled antibody and reduction of the obtained value of the IF 40. However, as Voss *et al.* also revealed an IF of only 70% for radiolabeled ch14.18 using immobilized GD2 22, the low cell surface reactivity might also be influenced by modification of an accessible amino acid either within or close to the antigen binding site of ch14.18/CHO and its related antibodies. Indeed, CDR2 of the parental 14G2a light chain contains a lysine residue at position 55 that is involved in recognition of GD2 41. Consequently, lysine coupling might not be ideal for radiolabeling GD2-specific antibodies, and further investigation of linker chemistry or purification of the radiolabeled antibodies might be helpful in improving the quality of GD2-specific radioimmunoconjugates. The use of derivatives such as maleimide DOTA or NH_2_-DOTA-GA offers other potential conditions for conjugation 42, 43 that might minimize the loss of immunoreactivity. Vavere *et al*. 23 radiolabeled the humanized variant of ch14.18 (hu14.18K322A) using p-NH_2_-Bn-DOTA, which binds to glutamic acid residues and showed that target binding is retained. The same group also used p-SCN-Bn-NOTA to radiolabel hu14.18K322A 24, unfortunately for both immunoconjugates the immunoreactive fraction is not reported or compared. Nevertheless, also other conjugation strategies could reduce immunoreactivity as binding site of ch14.18/CHO also contains glutamic and aspartic acid (heavy chain E101, D52). Thus, additional conjugation routes and quality control assays have to be explored in future work.

In our preclinical *in vivo* PET studies, [^64^Cu]Cu-DOTAGA-ch14.18/CHO revealed high tumor accumulation with a %ID/g of 31.6 ± 5.8; this is at least on par with other radiolabeled antibodies such as HER2/neu-specific trastuzumab 44 and EGFR-specific cetuximab 45. The difference of a factor 2-2.5 that was observed between *in vivo* and *ex vivo* quantification is a phenomenon that has been described previously and is mainly caused by the influence of different complex factors including scatter and its correction methods, attenuation as well as the partial volume effects in the reconstructed VOI, while the gamma counter is not influenced by this as it does not involve any information about spatial distribution of the measured radioactivity 46, 47. However, for the scope of this manuscript the relative differences between the NB and the control tumors as well as the blocked and the non-blocked experimental animals is more important. These relative differences are largely consistent throughout both methods.

Although the immunoreactivity of our antibody seemed to be impaired by radiolabeling, both the NB uptake of ch14.18/CHO and the TMRs significantly exceeded the values obtained for the control antibody, indicating specific tumor uptake of [^64^Cu]Cu-DOTAGA-ch14.18/CHO. *In vivo* blocking studies and the significant difference in NB to control tumor uptake of the radiolabeled antibody further support its *in vivo* specificity. Full tumor saturation was not reached by the blocking dose, however as the blocking experiment was performed to verify specificity, titration of the full blocking dose was not in scope of this manuscript.

In a study by Voss *et al*., ^64^Cu-SarAr-labeled ch14.18 showed a lower xenograft uptake compared to our findings, although the reported IF was higher 22. However, tumor accumulation is highly influenced by tissue characteristics such as target expression 24, interstitial pressure 48, vessel leakage 49, and necrosis 50, 51, and potential differences in these characteristics decrease the comparability of different tumor models.

To facilitate translation to the clinic and to allow for labeling with therapeutic isotopes such as ^177^Lu, DOTAGA, a derivative of DOTA which is widely used as a chelator in clinical radiopharmaceuticals, was used in our preclinical studies. However, a reported disadvantage of the use of DOTA derivatives is the loss of copper *in vivo* with subsequent accumulation of unbound activity in the liver 52. Beside the liver, free ^64^Cu is accumulating in muscle, kidneys as well as to a lesser extent in the red bone marrow of patients revealed by PET imaging with [^64^Cu]CuCl_2_ 53. For safety reasons, we radiolabeled ch14.18/CHO with NOTA for the first patient 26, 54, which has been reported to have increased complexation stability for ^64^Cu compared to DOTA and is also used clinically.

In addition to NOTA, many other bifunctional chelators have been examined in preclinical studies to e.g. improve stability 22, 54. For instance, ch14.18 has previously been radiolabeled using the newly designed chelator 1-N-(4-aminobenzyl)-3,6,10,13,16,19-hexaazabicyclo[6.6.6]-eicosane-1,8-diamine) (SarAr), which is based on a diamsar ligand 22, 55. However, the lack of human safety data for SarAr precludes its immediate clinical translation. Furthermore, diamsar-based chelators and smaller chelators such as NOTA are not suitable for use with complex radionuclei with larger diameters such as ^177^Lu 42, which is disadvantageous in regards of our future aim to evaluate the combination of ^64^Cu-ImmunoPET and ^177^Lu based radioimmunotherapy.

All of the radioconjugates prepared in our study showed high stability *in vitro* and preclinically less than 8% of the injected activity was found per gram liver in the used experimental tumor models* in vivo*, a level that is comparable to the liver accumulation that was quantified for ch14.18 radiolabeled using SarAr. For this reason, we believe that DOTAGA-labeled GD2-specific antibodies are currently an adequate choice for the development of clinical approaches, especially when their use in radioimmunotherapy is the primary goal.

In our first translational approach, a patient suffering from NB metastases was investigated by [^64^Cu]Cu-NOTA-ch14.18/CHO PET/MRI before and after immunotherapy. Through the use of ImmunoPET we were able to identify NB metastases and potentially to predict their response to immunotherapy, as well as to detect disease progression confirmed by the clinical standard methods MRI and [^123^I]MIBG scintigraphy. In addition to NB uptake, our PET data indicate presence of [^64^Cu]Cu-NOTA-ch14.18/CHO in the blood pool as well as most probably a perfusion-dependent accumulation in different organs and bone marrow which is in line with the distribution of other antibody constructs 38, 56. As this background accumulation might reduce the sensitivity in lesion detection, later imaging e.g. at 48 h will be evaluated in the future. The choice of 24 hours after injection for imaging is based on our preclinical findings and on a practicable clinical workflow, especially for the diagnosis of children. Allowing a slightly longer uptake time between injection and imaging might decrease the amount of radioactivity in the blood and would still be possible with ^64^Cu. Smaller GD2-specific constructs, such as antibody fragments, might have better accessibility and would allow for earlier imaging; however, for our aim of enabling image-guided therapy it was essential to use exactly the same biological substance in imaging and therapy.

Interestingly, a femoral lesion with prominent [^64^Cu]Cu-NOTA-ch14.18/CHO uptake in the baseline scan showed strong response to immunotherapy as assessed by MRI and [^123^I]MIBG scintigraphy after the third treatment cycle. The high uptake of the radiolabeled antibody at baseline might indicate very good accessibility of the therapeutic antibody to the lesion and an increased likelihood of response to the treatment. On the contrary, there was a lack of uptake of the radiolabeled antibody by other lesions identified in [^123^I]MIBG scans or the MRI at baseline. In reverse conclusion, this finding possibly indicates restricted access to the therapeutic antibody and potentially gives very valuable predictions regarding a limited treatment efficacy of a corresponding immunotherapy. Potential reasons for a lack of accumulation might be a reduction in GD2 expression or restriction of access due to perfusion or diffusion effects. In addition, the use of a non-optimal dose of the radiolabeled antibody might decrease its accumulation 56; thus, a phase I dose escalation clinical trial is needed. Furthermore, the potential impact of the time interval between the [^123^I]MIBG and GD2-PET scans must be considered.

Follow-up GD2-PET scanning was performed 2.5 weeks after the last treatment with the therapeutic antibody to verify the imaging capabilities of [^64^Cu]Cu-NOTA-ch14.18/CHO and to compare signs of response in GD2-PET to standard methods. At this time point, the femoral lesion showed a reduced uptake of [^64^Cu]Cu-NOTA-ch14.18/CHO. Saturation of GD2 receptors is unlikely since the antibody has a half-life of 3.2 days in serum 9. Treatment antibody bound to the tumor significantly after the unbound antibody is cleared from the blood at the time of the follow up study is unlikely due to target turn over, shedding and internalization. As we see also lesions with increasing [^64^Cu]Cu-NOTA-ch14.18/CHO uptake in the follow-up PET compared to the baseline study, blocking might not explain the decreasing uptake in the lesions e.g. in the right femur.

The identification of non-responding lesions that were detected at baseline or of new lesions in a follow-up GD2-PET after treatment might indicate an insufficient treatment dose or disease progression. Thus, in image-guided immunotherapy, it is important to choose the right therapeutic dose 57, identify potentially inaccessible lesions and optimize the time points during the course of disease for therapeutic antibody application 58.

The appearance of lesions in the [^123^I]MIBG or MRI scans but not in the GD2-PET scan should be further addressed in prospective clinical studies. Although [^123^I]MIBG reveals NB with limited sensitivity, its specificity is high 59. T2-weighted MRI exhibits a high sensitivity of detection of NB but a low specificity, although this might be improved by the use of diffusion weighted (DW) MRI. Thus, GD2-PET could potentially be useful not only for guiding antibody-based GD2 immunotherapy but also in combination with DW-MRI to improve the sensitivity and specificity of detection of NB lesions.

## Conclusion

The focus of our study was the establishment of dedicated theranostic imaging for GD2 directed treatments with the ultimate goal of guiding therapy for NB. Using the clinically approved antibody ch14.18/CHO, we developed a chelator- and radiolabeling protocol with immediate translational potential and produced a radioimmunoconjugate that shows specific tumor targeting *in vivo*, high NB uptake, and low background accumulation. Finally, the potential clinical relevance of GD2-ImmunoPET was shown in a first-in-man compassionate use imaging trial. Without doubt, larger trials are needed to explore the clinical characteristics and limitations of this approach. However, these first results provide insight into a new method that offers the potential for *a priori* treatment response estimation for GD2 directed (radio)immunotherapy.

## Supplementary Material

Supplementary figures.

## Figures and Tables

**Table 1:** ***Ex vivo* biodistribution of radiolabeled antibodies.** The* ex vivo* biodistribution of [^64^Cu]Cu-DOTAGA-ch14.18/CHO with and without blocking of GD2 using 500 µg unlabeled ch14.18/CHO 24 h prior to tracer administration was quantified 48 h after tracer injection. The distribution of a nonspecific control antibody (control ab) at 48 h is shown as a reference. Values are given in % ID/g.

	[^64^Cu]CuDOTAGA-ch14.18/CHO	[^64^Cu]CuDOTAGA-control ab	[^64^Cu]CuDOTAGA-ch14.18/CHO Block
Neuroblastoma	31.57 ± 5.79	3.71 ± 0.74	22.73 ± 1.95
Control tumor	6.94 ± 2.79	-	-
Blood	7.81 ± 1.85	6.43 ± 1.64	7.95 ± 0.70
Heart	2.78 ± 0.39	2.98 ± 0.26	2.93 ± 0.50
Lung	5.78 ± 1.71	6.68 ± 2.31	5.46 ± 2.09
Liver	7.33 ± 1.44	12.17 ± 1.12	5.71 ± 0.80
Kidney	4.51 ± 0.87	5.91 ± 0.41	4.16 ± 0.74
Muscle	1.06 ± 0.19	0.91 ± 0.07	1.20 ± 0.26
TMR (NB)	30.15 ± 5.00	4.08 ± 0.81	20.13 ± 5.56
TLR (NB)	4.37 ± 0.72	0.31 ± 0.08	4.07 ± 0.73
TBR (NB)	4.13 ± 0.62	0.60 ± 0.18	2.88 ± 0.28

## Abbreviations

ACR: antibody-to-chelator ratios
AMG: Medicinal Products Act (“Arzneimittelgesetz”)
BSA: bovine serum albumin
CHO: chinese hamster ovary cells
DOTA: 1,4,7,10-tetraazacyclododecane-1,4,7,10-tetraacetic acid
DOTAGA: 2-(4,7,10-tris(carboxymethyl)-1,4,7,10-tetraazacyclododecan-1-yl)pentanedioic acid
DTPA: diethylenetriaminepentaacetic acid
DTT: dithiothreitol
DWI: transverse diffusion-weighted imaging
EDTA: ethylenediaminetetraacetic acid
ESI: electrospray ionization
FCS: fetal calf serum
GMP: good manufacturing practice
H&E: hematoxylin and eosin
HEK-293: human embryonic kidney cells
HEPES: 4-(2-hydroxyethyl)-1-piperazineethanesulfonic acid
HPSEC: high performance size exclusion chromatography
ID/cc: injected dose per cubic centimeter
IF: immunoreactive fraction
LC: liquid chromatography
MIBG: metaiodobenzylguanidine
MRI: magnetic resonance imaging
NB: neuroblastoma
NCS: isothiocyanate
NOTA: 1,4,7-triazacyclononane-1,4,7-triacetic acid
PET: positron emission tomography
RCP: radiochemical purity
RIT: radioimmunotherapy
RT: room temperature
SarAr: 1-N-(4-aminobenzyl)-3,6,10,13,16,19-hexaazabicyclo[6.6.6]-eicosane-1,8-diamine)
SIOPEN: Society of Pediatric Oncology European Neuroblastoma Group
SPECT: single-photon emission computed tomography
TBR: tumor-to-blood ratio
TIRM: T2 turbo inversion recovery magnitude
TLC: thin-layer chromatography
TLR: tumor-to-liver ratio
TMR: tumor-to-muscle ratio

## References

[1] Maris JM, Hogarty MD, Bagatell R, Cohn SL (2007). Neuroblastoma. Lancet.

[2] Whittle SB, Smith V, Doherty E, Zhao S, McCarty S, Zage PE (2017). Overview and recent advances in the treatment of neuroblastoma. Expert Rev Anticancer Ther.

[3] Navid F, Santana VM, Barfield RC (2010). Anti-GD2 antibody therapy for GD2-expressing tumors. Curr Cancer Drug Targets.

[4] Ploessl C, Pan A, Maples KT, Lowe DK (2016). Dinutuximab: An Anti-GD2 Monoclonal Antibody for High-Risk Neuroblastoma. Ann Pharmacother.

[5] Zeng Y, Fest S, Kunert R, Katinger H, Pistoia V, Michon J (2005). Anti-neuroblastoma effect of ch14.18 antibody produced in CHO cells is mediated by NK-cells in mice. Mol Immunol.

[6] Borys MC, Dalal NG, Abu-Absi NR, Khattak SF, Jing Y, Xing Z (2010). Effects of culture conditions on N-glycolylneuraminic acid (Neu5Gc) content of a recombinant fusion protein produced in CHO cells. Biotechnol Bioeng.

[7] Taylor RE, Gregg CJ, Padler-Karavani V, Ghaderi D, Yu H, Huang S (2010). Novel mechanism for the generation of human xeno-autoantibodies against the nonhuman sialic acid N-glycolylneuraminic acid. J Exp Med.

[8] Shepherd AJ, Wilson NJ, Smith KT (2003). Characterisation of endogenous retrovirus in rodent cell lines used for production of biologicals. Biologicals.

[9] Ladenstein R, Weixler S, Baykan B, Bleeke M, Kunert R, Katinger D (2013). Ch14.18 antibody produced in CHO cells in relapsed or refractory Stage 4 neuroblastoma patients: a SIOPEN Phase 1 study. MAbs.

[10] Siebert N, Eger C, Seidel D, Juttner M, Zumpe M, Wegner D (2016). Pharmacokinetics and pharmacodynamics of ch14.18/CHO in relapsed/refractory high-risk neuroblastoma patients treated by long-term infusion in combination with IL-2. MAbs.

[11] Keyel ME, Reynolds CP (2019). Spotlight on dinutuximab in the treatment of high-risk neuroblastoma: development and place in therapy. Biologics.

[12] Richards RM, Sotillo E, Majzner RG (2018). CAR T Cell Therapy for Neuroblastoma. Front Immunol.

[13] Kramer K, Kushner BH, Modak S, Pandit-Taskar N, Smith-Jones P, Zanzonico P (2010). Compartmental intrathecal radioimmunotherapy: results for treatment for metastatic CNS neuroblastoma. J Neurooncol.

[14] Kramer K, Pandit-Taskar N, Humm JL, Zanzonico PB, Haque S, Dunkel IJ (2018). A phase II study of radioimmunotherapy with intraventricular (131) I-3F8 for medulloblastoma. Pediatr Blood Cancer.

[15] Kramer K, Humm JL, Souweidane MM, Zanzonico PB, Dunkel IJ, Gerald WL (2007). Phase I study of targeted radioimmunotherapy for leptomeningeal cancers using intra-Ommaya 131-I-3F8. J Clin Oncol.

[16] Mueller WP, Coppenrath E, Pfluger T (2013). Nuclear medicine and multimodality imaging of pediatric neuroblastoma. Pediatr Radiol.

[17] Zhang Y, Kupferschlaeger J, Lang P, Reischl G, Handgretinger RJ, Fougere C (2022). (131)I-GD2-ch14.18 Scintigraphy to Evaluate Option for Radioimmunotherapy in Patients with Advanced Tumors. J Nucl Med.

[18] Schumacher-Kuckelkorn R, Volland R, Gradehandt A, Hero B, Simon T, Berthold F (2017). Lack of immunocytological GD2 expression on neuroblastoma cells in bone marrow at diagnosis, during treatment, and at recurrence. Pediatr Blood Cancer.

[19] Jain RK (1990). Physiological barriers to delivery of monoclonal antibodies and other macromolecules in tumors. Cancer Res.

[20] Yeh SD, Larson SM, Burch L, Kushner BH, Laquaglia M, Finn R (1991). Radioimmunodetection of neuroblastoma with iodine-131-3F8: correlation with biopsy, iodine-131-metaiodobenzylguanidine and standard diagnostic modalities. J Nucl Med.

[21] Reuland P, Geiger L, Thelen MH, Handgretinger R, Haase B, Muller-Schauenburg W (2001). Follow-up in neuroblastoma: comparison of metaiodobenzylguanidine and a chimeric anti-GD2 antibody for detection of tumor relapse and therapy response. J Pediatr Hematol Oncol.

[22] Voss SD, Smith SV, DiBartolo N, McIntosh LJ, Cyr EM, Bonab AA (2007). Positron emission tomography (PET) imaging of neuroblastoma and melanoma with 64Cu-SarAr immunoconjugates. Proc Natl Acad Sci U S A.

[23] Vavere AL, Butch ER, Dearling JL, Packard AB, Navid F, Shulkin BL (2012). 64Cu-p-NH2-Bn-DOTA-hu14.18K322A, a PET radiotracer targeting neuroblastoma and melanoma. J Nucl Med.

[24] Butch ER, Mead PE, Amador Diaz V, Tillman H, Stewart E, Mishra JK (2018). Positron Emission Tomography Detects In Vivo Expression of Disialoganglioside GD2 in Mouse Models of Primary and Metastatic Osteosarcoma. Cancer Research. 2019: canres.3340.

[25] Maier FC, Schmitt J, Maurer A, Ehrlichmann W, Reischl G, Nikolaou K (2016). Correlation between positron emission tomography and Cerenkov luminescence imaging in vivo and ex vivo using 64Cu-labeled antibodies in a neuroblastoma mouse model. Oncotarget.

[26] Roosenburg S, Laverman P, Joosten L, Cooper MS, Kolenc-Peitl PK, Foster JM (2014). PET and SPECT imaging of a radiolabeled minigastrin analogue conjugated with DOTA, NOTA, and NODAGA and labeled with (64)Cu, (68)Ga, and (111)In. Mol Pharm.

[27] Seidel UJ, Schlegel P, Grosse-Hovest L, Hofmann M, Aulwurm S, Pyz E (2016). Reduction of Minimal Residual Disease in Pediatric B-lineage Acute Lymphoblastic Leukemia by an Fc-optimized CD19 Antibody. Mol Ther.

[28] Spycher PR, Amann CA, Wehrmuller JE, Hurwitz DR, Kreis O, Messmer D (2017). Dual, Site-Specific Modification of Antibodies by Using Solid-Phase Immobilized Microbial Transglutaminase. Chembiochem.

[29] Lewis MR, Wang M, Axworthy DB, Theodore LJ, Mallet RW, Fritzberg AR (2003). In vivo evaluation of pretargeted 64Cu for tumor imaging and therapy. J Nucl Med.

[30] Lindmo T, Bunn PA Jr (1986). Determination of the true immunoreactive fraction of monoclonal antibodies after radiolabeling. Methods Enzymol.

[31] Cooper MS, Sabbah E, Mather SJ (2006). Conjugation of chelating agents to proteins and radiolabeling with trivalent metallic isotopes. Nat Protoc.

[32] Hofmann M, Bezrukov I, Mantlik F, Aschoff P, Steinke F, Beyer T (2011). MRI-based attenuation correction for whole-body PET/MRI: quantitative evaluation of segmentation- and atlas-based methods. J Nucl Med.

[33] Gatidis S, Schmidt H, Gucke B, Bezrukov I, Seitz G, Ebinger M (2016). Comprehensive Oncologic Imaging in Infants and Preschool Children With Substantially Reduced Radiation Exposure Using Combined Simultaneous (1)(8)F-Fluorodeoxyglucose Positron Emission Tomography/Magnetic Resonance Imaging: A Direct Comparison to (1)(8)F-Fluorodeoxyglucose Positron Emission Tomography/Computed Tomography. Invest Radiol.

[34] Schaefer JF, Vollmar J, Schick F, Vonthein R, Seemann MD, Aebert H (2004). Solitary pulmonary nodules: dynamic contrast-enhanced MR imaging-perfusion differences in malignant and benign lesions. Radiology.

[35] Bezrukov I, Schmidt H, Gatidis S, Mantlik F, Schafer JF, Schwenzer N (2015). Quantitative Evaluation of Segmentation- and Atlas-Based Attenuation Correction for PET/MR on Pediatric Patients. J Nucl Med.

[36] Ahmed M, Cheung NK (2014). Engineering anti-GD2 monoclonal antibodies for cancer immunotherapy. FEBS Lett.

[37] Cheung NK, Ostrovnaya I, Kuk D, Cheung IY (2015). Bone marrow minimal residual disease was an early response marker and a consistent independent predictor of survival after anti-GD2 immunotherapy. J Clin Oncol.

[38] Bensch F, van der Veen EL, Lub-de Hooge MN, Jorritsma-Smit A, Boellaard R, Kok IC (2018). (89)Zr-atezolizumab imaging as a non-invasive approach to assess clinical response to PD-L1 blockade in cancer. Nat Med.

[39] Garrow AA, Andrews JPM, Gonzalez ZN, Corral CA, Portal C, Morgan TEF (2020). Preclinical dosimetry models and the prediction of clinical doses of novel positron emission tomography radiotracers. Sci Rep.

[40] Li RX, Ladisch S (1991). Shedding of human neuroblastoma gangliosides. Biochim Biophys Acta.

[41] Horwacik I, Golik P, Grudnik P, Kolinski M, Zdzalik M, Rokita H (2015). Structural Basis of GD2 Ganglioside and Mimetic Peptide Recognition by 14G2a Antibody. Mol Cell Proteomics.

[42] Ramogida CF, Orvig C (2013). Tumour targeting with radiometals for diagnosis and therapy. Chem Commun (Camb).

[43] Zeglis BM, Lewis JS (2011). A practical guide to the construction of radiometallated bioconjugates for positron emission tomography. Dalton Trans.

[44] Paudyal P, Paudyal B, Hanaoka H, Oriuchi N, Iida Y, Yoshioka H (2010). Imaging and biodistribution of Her2/neu expression in non-small cell lung cancer xenografts with Cu-labeled trastuzumab PET. Cancer Sci.

[45] Ping Li W, Meyer LA, Capretto DA, Sherman CD, Anderson CJ (2008). Receptor-binding, biodistribution, and metabolism studies of 64Cu-DOTA-cetuximab, a PET-imaging agent for epidermal growth-factor receptor-positive tumors. Cancer Biother Radiopharm.

[46] Wiehr S, Buhler P, Gierschner D, Wolf P, Rolle AM, Kesenheimer C (2014). Pharmacokinetics and PET imaging properties of two recombinant anti-PSMA antibody fragments in comparison to their parental antibody. Prostate.

[47] Mannheim JG, Judenhofer MS, Schmid A, Tillmanns J, Stiller D, Sossi V (2012). Quantification accuracy and partial volume effect in dependence of the attenuation correction of a state-of-the-art small animal PET scanner. Phys Med Biol.

[48] Wu M, Frieboes HB, Chaplain MA, McDougall SR, Cristini V, Lowengrub JS (2014). The effect of interstitial pressure on therapeutic agent transport: coupling with the tumor blood and lymphatic vascular systems. J Theor Biol.

[49] Dvorak HF, Nagy JA, Feng D, Brown LF, Dvorak AM (1999). Vascular permeability factor/vascular endothelial growth factor and the significance of microvascular hyperpermeability in angiogenesis. Curr Top Microbiol Immunol.

[50] Tabrizi M, Bornstein GG, Suria H (2010). Biodistribution mechanisms of therapeutic monoclonal antibodies in health and disease. AAPS J.

[51] Jain RK (2012). Delivery of molecular and cellular medicine to solid tumors. Adv Drug Deliv Rev.

[52] Stasiuk GJ, Long NJ (2013). The ubiquitous DOTA and its derivatives: the impact of 1,4,7,10-tetraazacyclododecane-1,4,7,10-tetraacetic acid on biomedical imaging. Chem Commun (Camb).

[53] Righi S, Ugolini M, Bottoni G, Puntoni M, Iacozzi M, Paparo F (2018). Biokinetic and dosimetric aspects of (64)CuCl2 in human prostate cancer: possible theranostic implications. EJNMMI Res.

[54] Dearling JL, Voss SD, Dunning P, Snay E, Fahey F, Smith SV (2011). Imaging cancer using PET-the effect of the bifunctional chelator on the biodistribution of a (64)Cu-labeled antibody. Nucl Med Biol.

[55] Dearling JL, Paterson BM, Akurathi V, Betanzos-Lara S, Treves ST, Voss SD (2015). The ionic charge of copper-64 complexes conjugated to an engineered antibody affects biodistribution. Bioconjug Chem.

[56] Dijkers EC, Oude Munnink TH, Kosterink JG, Brouwers AH, Jager PL, de Jong JR (2010). Biodistribution of 89Zr-trastuzumab and PET imaging of HER2-positive lesions in patients with metastatic breast cancer. Clin Pharmacol Ther.

[57] Jauw YWS, Huisman MC, Nayak TK, Vugts DJ, Christen R, Naegelen VM (2018). Assessment of target-mediated uptake with immuno-PET: analysis of a phase I clinical trial with an anti-CD44 antibody. EJNMMI Res.

[58] Jauw YW, Menke-van der Houven van Oordt CW, Hoekstra OS, Hendrikse NH, Vugts DJ, Zijlstra JM (2016). Immuno-Positron Emission Tomography with Zirconium-89-Labeled Monoclonal Antibodies in Oncology: What Can We Learn from Initial Clinical Trials?. Front Pharmacol.

[59] Pfluger T, Schmied C, Porn U, Leinsinger G, Vollmar C, Dresel S (2003). Integrated imaging using MRI and 123I metaiodobenzylguanidine scintigraphy to improve sensitivity and specificity in the diagnosis of pediatric neuroblastoma. AJR Am J Roentgenol.

