# Supplementary material for: Translational immunoPET imaging using a radiolabeled GD2-specific antibody in neuroblastoma
Source: Theranostics. 2022 Jul 18;12(13):5615–30. doi: 10.7150/thno.56736 (PMC9373823; doi:10.7150/thno.56736)
Supplement: Supplementary file 1 — Supplementary figures. [file thnov12p5615s1.pdf]

Supplementary data

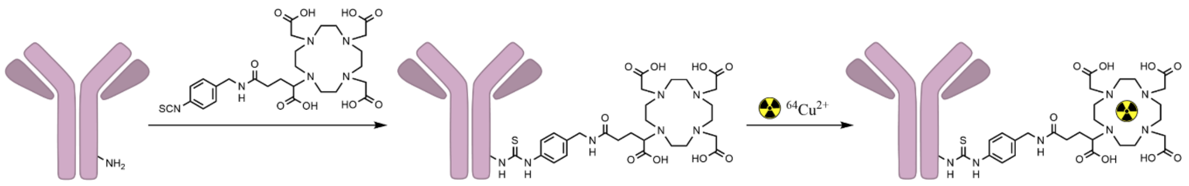

Figure S1: Scheme of the radiolabeling procedure
Schematic representation of chelator conjugation and radiolabeling of ch14.18. Chelator is first conjugated to lysine side chains (amines, NH2) through its isothiocyanate moiety (SCN). After removal of excess chelator, the buffered radioisotope ^{64}Cu is bound to the chelator.

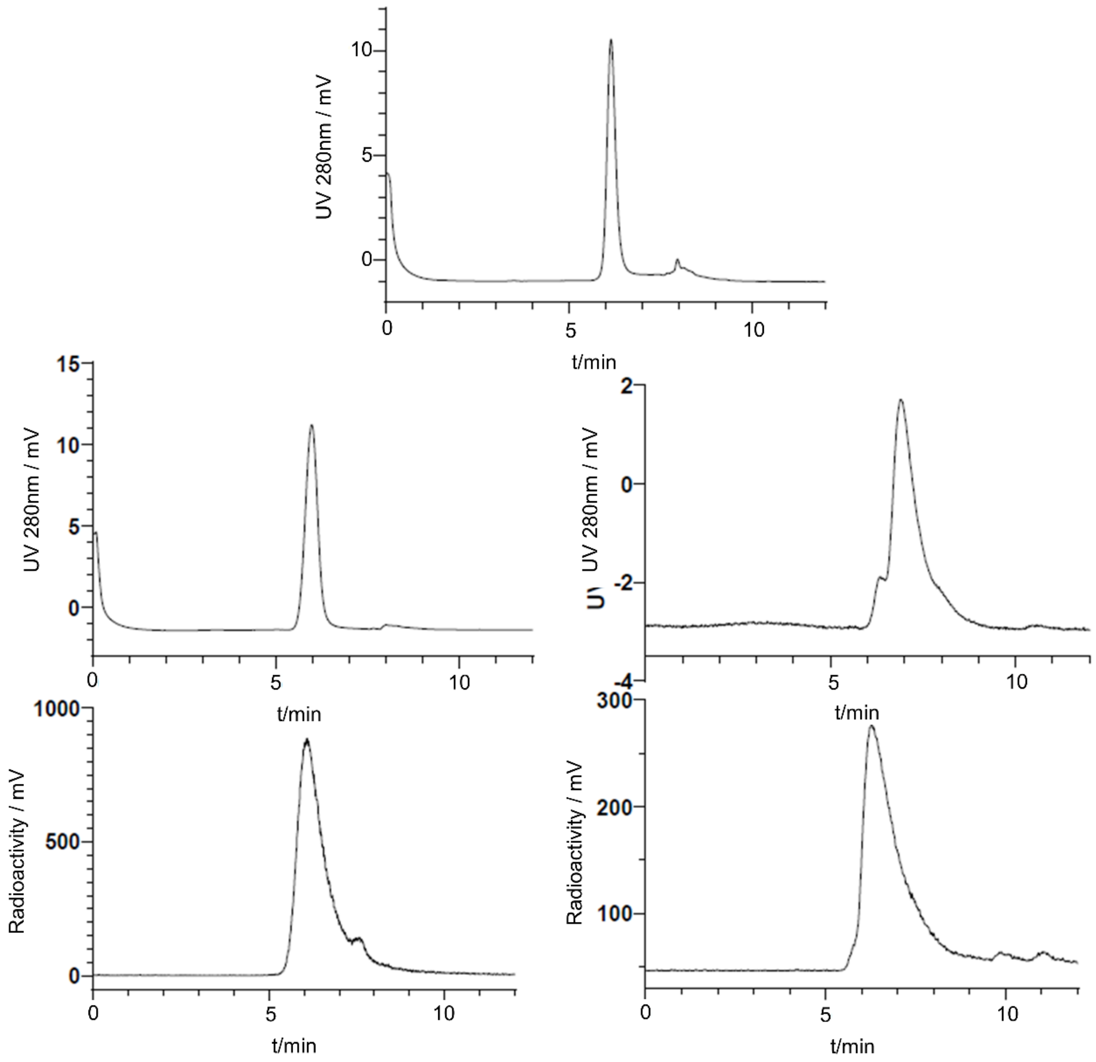

Figure S2: Chromatograms of the conjugates

Representative chromatograms of ch14.18 before (upper row) and after conjugation (middle row, left: DOTAGA, right: NOTA) and radiolabeling (lower row, left: DOTAGA, right: NOTA). UV traces at 280 nm or radioactivity traces are shown for non-radioactive or radiolabeled proteins, respectively.

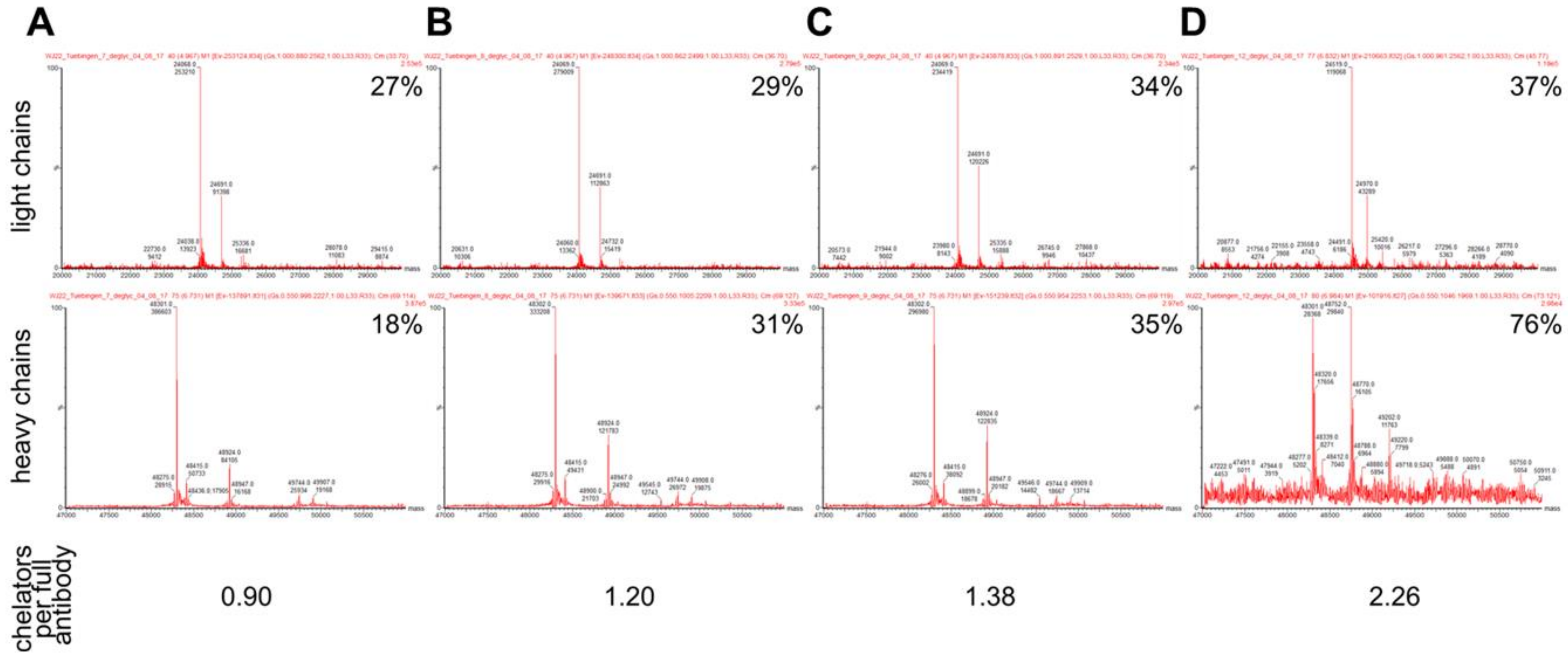

Figure S3: Mass spectra of the chelator-conjugates
Mass spectra of heavy and light chains of chelator-conjugated antibodies. A: DOTAGA-ch14.18 conjugated with 1:5 molar ratio Ab:chelator; B: dto, 1:10; C: dto, 1:15; D: NOTA-ch14.18 for human use.

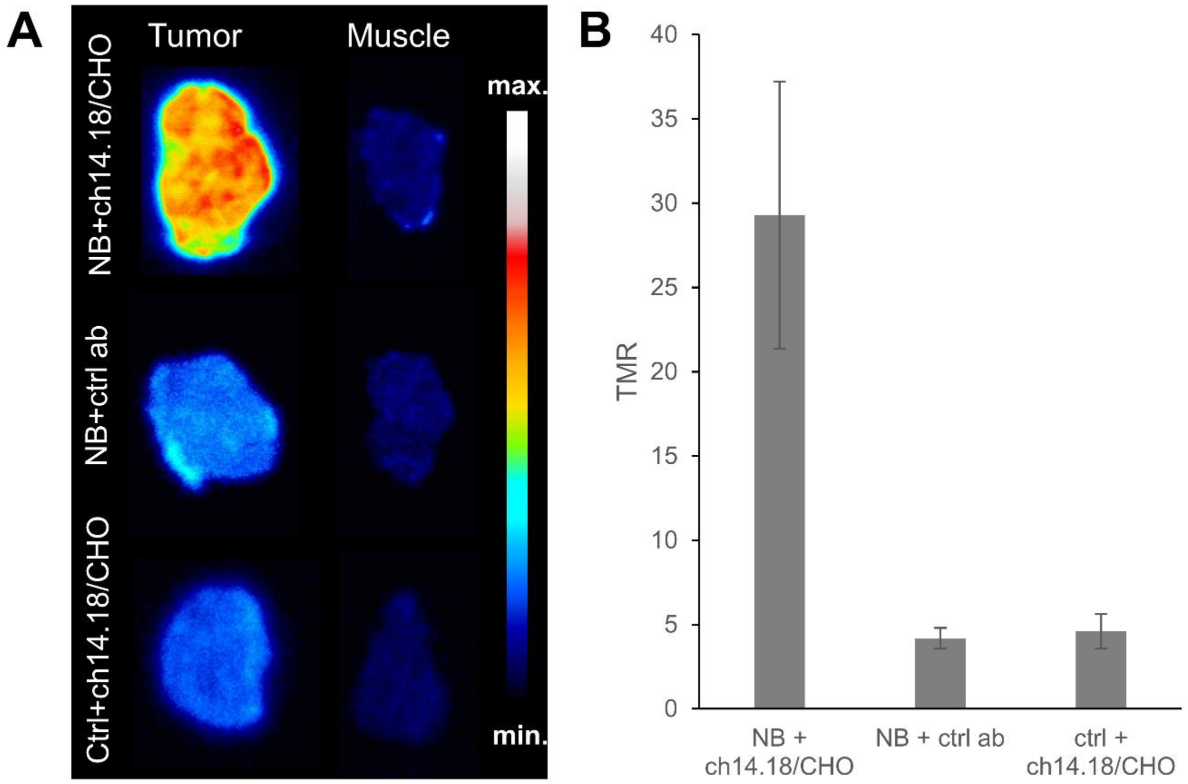

Figure S4: Autoradiography of tissue samples
Autoradiography acquired from tumor slices after the 48 h imaging time point show a highly increased signal in the NB after injection of radiolabeled ch14.18/CHO compared to controls (NB + control ab or control tumor + radiolabeled ch14.18/CHO). Muscle tissue is shown as reference and TMR are quantified for comparison between studies.

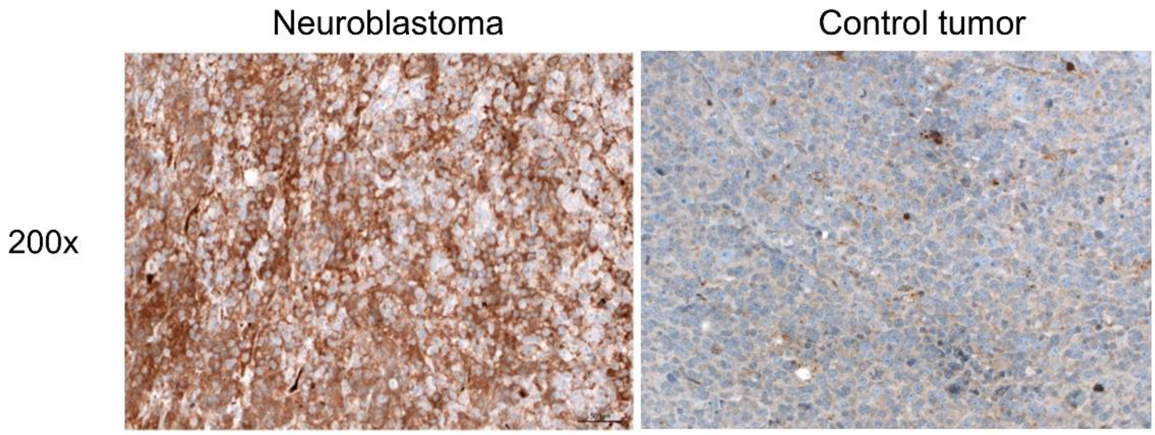

Figure S5: ***GD2 staining of tumor sections***
GD2 expression in the tumor tissue was verified using the GD2-specific antibody. GD2 expression was only observed in NBs.

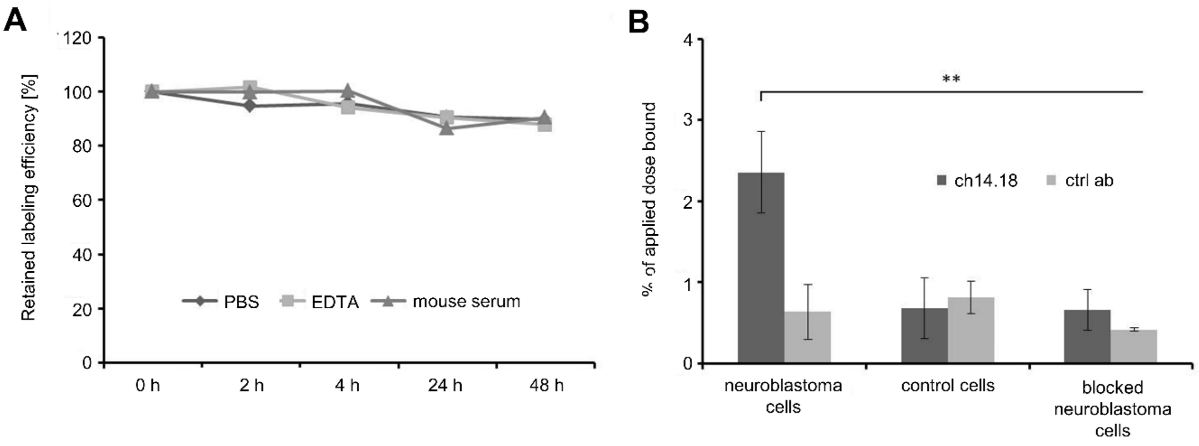

Figure S6: *In vitro* testing of the clinically ^{64}Cu-labeled ch14.18/CHO

Stability and specificity of ch14.18/CHO, which was chelator- and radiolabeled under GMP conditions, was tested *in vitro*. The radioconjugation was verified to be stable over 48 h in PBS, EDTA and mouse serum via TLC (A) and [^{64}Cu]Cu-p-SCN-Bn-NOTA-ch14.18 showed a specific binding to GD2 positive neuroblastoma cells (B).

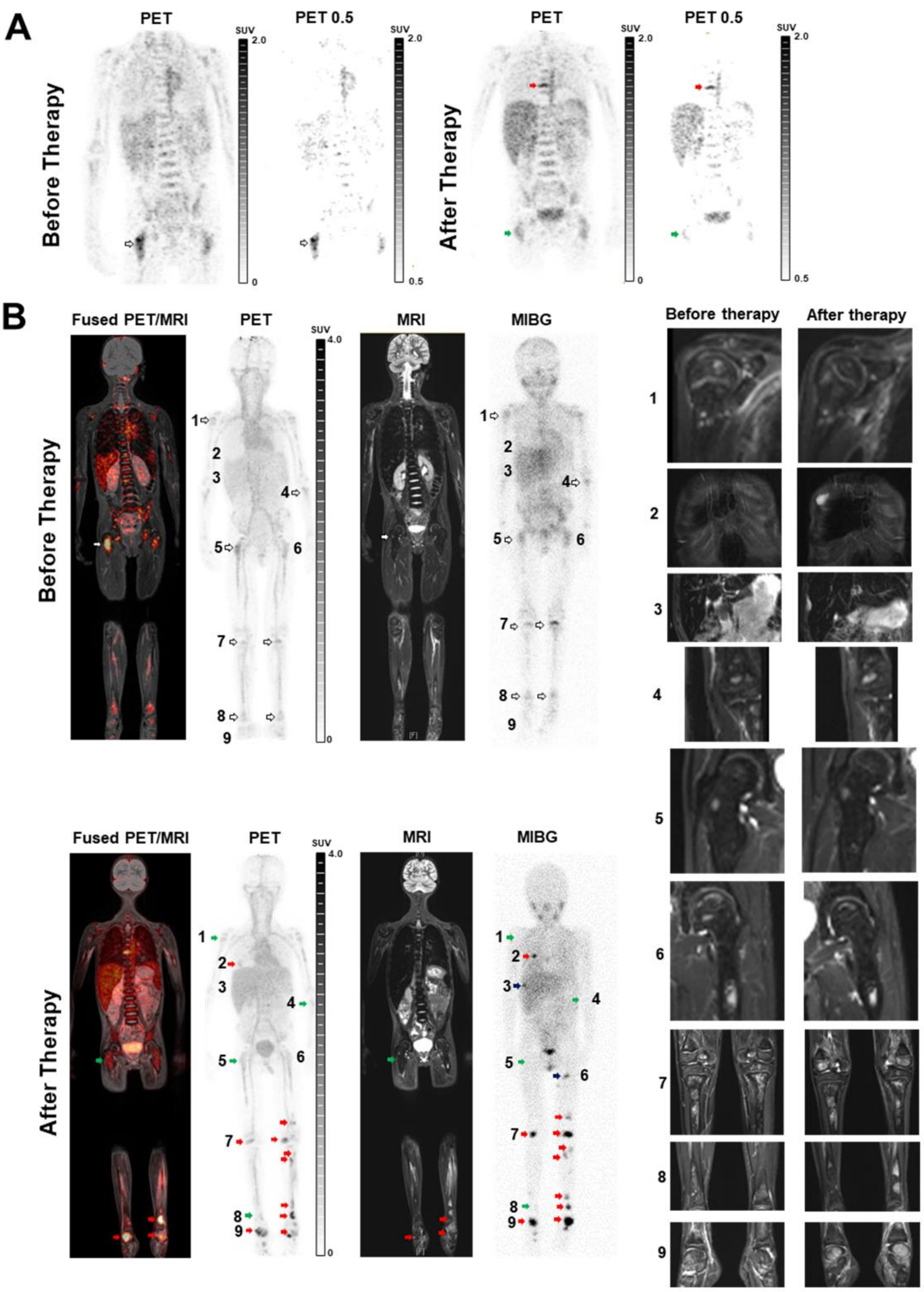

Figure S7: Clinical GD2-specific ImmunoPET/MRI of Neuroblastoma

A: To better distinguish between metastasis and the unspecific uptake in the bone (especially in the spine and the pelvis) the windowing was adapted to minimize the visible background signal (PET 0.5). A new lesion in the spine was indicated by a red arrow, a lesions responding to treatment in the right femur was indicated by white and green arrows).

B: Exemplary lesions were numbered and the corresponding MRI (whole body T2 TIRM) slices were presented to highlight the morphogical changes: (1) right humerus: decreased intensity in the follow up MRI; (2) thorax right ventral: new bone lesion in the follow up MRI; (3) thorax right lateral: new bone lesion in the follow up MRI; (4) left elbow: lesions partly less, partly more intense; (5) right femur: no major changes; (6) left femur: no major changes; (7) right lateral femoral condyle: new bone lesion; left proximal tibia: new lesions; right tibia: no major changes; (8) right distal tibia: regressive lesions; left distal tibia: new lesions; (9) tarsus bilateral: new bone lesion.
